# Visible Light Promoted [3+2]-Cycloaddition for the Synthesis of Cyclopenta[*b*]chromenocarbonitrile Derivatives

**DOI:** 10.1021/acs.joc.3c02172

**Published:** 2023-11-21

**Authors:** Ewelina Kowalska, Mateusz Dyguda, Angelika Artelska, Anna Albrecht

**Affiliations:** † Institute of Organic Chemistry, Faculty of Chemistry, Lodz University of Technology, Żeromskiego 116, Łódź 90-924, Poland; ‡ Institute of Applied Radiation Chemistry, Lodz University of Technology, Żeromskiego 116, Łódź 90-924, Poland; § Institute of General and Ecological Chemistry, Faculty of Chemistry, Lodz University of Technology, Żeromskiego 116, Łódź 90-924, Poland

## Abstract

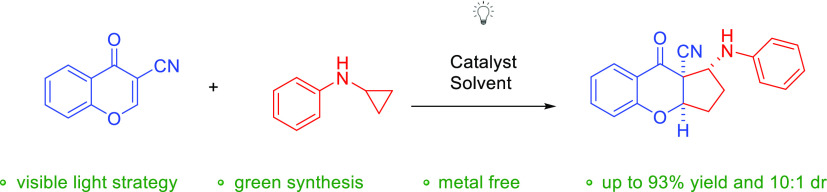

In the manuscript, a novel method for the preparation of cyclopenta[*b*]chromenocarbonitrile derivatives via [3+2] cycloaddition reaction of substituted 3-cyanochromones and *N*-cyclopropyloamines initiated by visible light catalysis has been described. The reaction was performed in the presence of Eosin Y as a photocatalyst. The key parameters responsible for the success of the described strategy are visible light, a small amount of photoredox catalyst, an anhydrous solvent, and an inert atmosphere.

## Introduction

**Scheme 1:**
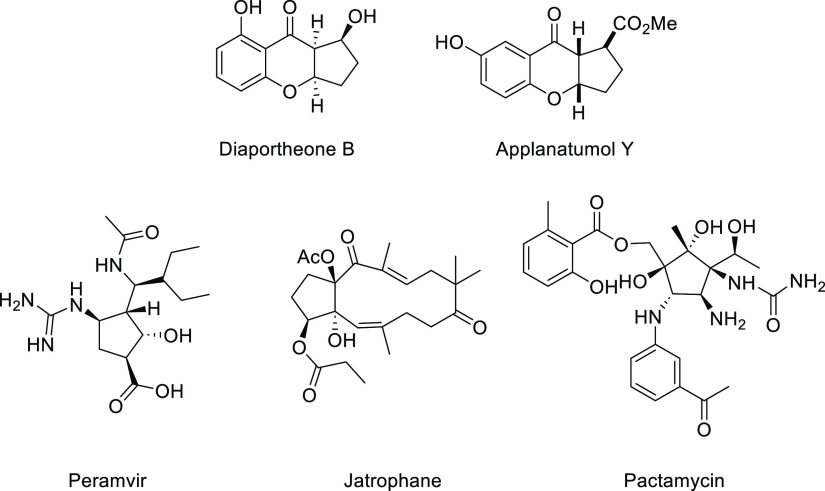
Biologically Relevant Cyclopentane Derivatives

Cyclopentachromene is a common structural motif that is present in many natural products. Selected examples of bioactive derivatives, relevant to the life-science industry, are shown in Scheme 1. For instance, the natural product diaportheone B was isolated from the endophytic fungus *Diaporthe* sp. P133 and possess antituberculosis activity against the virulent strain of *Mycobacterium tuberculosis*.^1^ Applanatumol Y was isolated by Cheng in 2016 from the fruiting body of *Ganoderma applanatum*, which is a wood-decaying fungi.^2^ Total synthesis of this compound was performed by Ito using a Morita–Baylis–Hillman reaction as a key step.^2b^ A cyclopentane structural motif constitutes a relevant building block that is widely employed in target-oriented synthesis and presented in many bioactive natural products and pharmaceuticals, including peramivir,^3^ prostaglandins,^4^ jatrophanes,^5^ and pactamycin.^6^

Visible-light-induced radical functionalization has emerged as a valuable tool in the toolbox of synthetic chemists, enabling the efficient and selective formation of carbon–carbon bonds and the creation of novel organic molecules. It continues to be an active area of research, with ongoing developments and applications across various fields.^7^ [3+2]-Cycloaddition reactions are a very useful tool for the synthesis of functionalized five-membered carbocycles or heterocycles.^8^ 1,3-Dipolar cycloaddition involving ionic intermediates constitutes the most popular example. Recently, a new approach to [3+2]-cycloadditions involving radical intermediates has been introduced.^9^

**Scheme 2:**
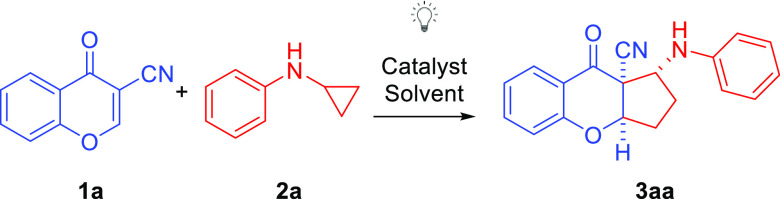
Objectives of Our Study

In continuation of our interest in the development of photocatalytic reactions,^10^ we turned our attention to the application of 3-cyanochromones and *N*-cyclopropyloamines as convenient starting materials for the synthesis of, interesting from a medicinal point of view, cyclopenta[*b*]chromenocarbonitrile products (Scheme 2).

Herein, we report an intermolecular [3+2]-cycloaddition of electron-poor olefins with cyclopropylanilines under visible light photocatalysis. The process is initiated by visible light, leading to the formation of cyclopentachromene derivatives. However, at the outset of our studies, challenges related to the radical nature of the transformation, its diastereoselectivity, and the utilization of trisubstituted olefin had to be considered.

## Results and Discussion

Initially, the [3+2]-photocycloaddition between chromen-4-one **5a** and *N*-cyclopropylaniline **2a** was performed in CH_2_Cl_2_ in the presence of *fac*-Ir(ppy)_3_ as a photocatalyst under irradiation with blue light and an inert atmosphere (Table 1, entry 1). As expected, no reaction was observed, which indicated the crucial role of the EWG-activation of **5** in the devised methodology. As highlighted in our previous work, the incorporation of an electron-withdrawing group into the chromenone is necessary to increase its electrophilic properties and hence for the reaction to occur. Therefore, the activation of **5** through the introduction of various activating groups in the 3-position was attempted (Table 1, entries 2–4). Most of the tested derivatives displayed no reactivity under these conditions, and the application of the CN group was crucial for the developed transformation (the cyano group exhibits a stronger electron-withdrawing effect when compared to other tested electron-deficient groups). Cycloaddition between 3-cyanochromone **1a** and *N*-cyclopropylaniline **2a** resulted in the formation of the desired product (Table 1, entry 4). The corresponding cyclopenta[*b*]chromeno-carbonitrile **3aa** was obtained with 57% yield as a mixture of two diastereoisomers, which differed in the configuration on the C-1 stereogenic center. The importance of the nitrile group for the developed reactivity is presumably related to the ability of this group to stabilize both radical and anionic intermediates.^11,12^ Various factors such as solvent, photocatalyst, and reaction concentration were tested in the course of optimization studies, while the temperature and *N*-cyclopropylaniline equivalents were held constant throughout. In the first part, the catalytic activity of five different photoredox catalysts was examined (with the irradiation with the light source of suitable wavelength) (Table 1, entries 4–8). All tested catalysts **4a–e** provided the desired reactivity with the best results obtained in the presence of Eosin Y (Table 1, entry 7). During further investigations, the effect of the solvent on the reaction outcome was evaluated (Table 1, entries 7 and 9–13). This part of the studies revealed dimethyl sulfoxide to be the best solvent, providing the target product in 82% yield and with the diastereoisomer ratio at a level of 5:1 (Table 1, entry 13). Change in the amount of the catalyst negatively affected reaction efficiency (Table 1, entries 13–15). Subsequently, the effect of the reaction concentration on both the yield and diastereoselectivity was evaluated, but neither increasing nor decreasing improved the results of the cycloaddition (Table 1, entries 13, 16, and 17). Eventually, cyclopenta[*b*]chromeno-carbonitrile **3aa** was formed in the presence of 5 mol% of Eosin Y in DMSO in 82% yield as a mixture of diastereoisomers in the 5:1 ratio. These results were validated on an initial scale up to 1.0 mmol, providing **3aa** in 75% yield (Table 1, entry 19). A series of control experiments demonstrated that the reaction did not take place in the absence of a photocatalyst or in the dark, indicating the crucial effect of a photoredox catalyst and the source of light on the reaction outcome (Table 1, entries 20 and 21). Finally, the experiment in the presence of TEMPO was carried out, and no reaction was observed, thus confirming the radical nature of the developed reaction (Table 1, entry 22).

**Table 1:**
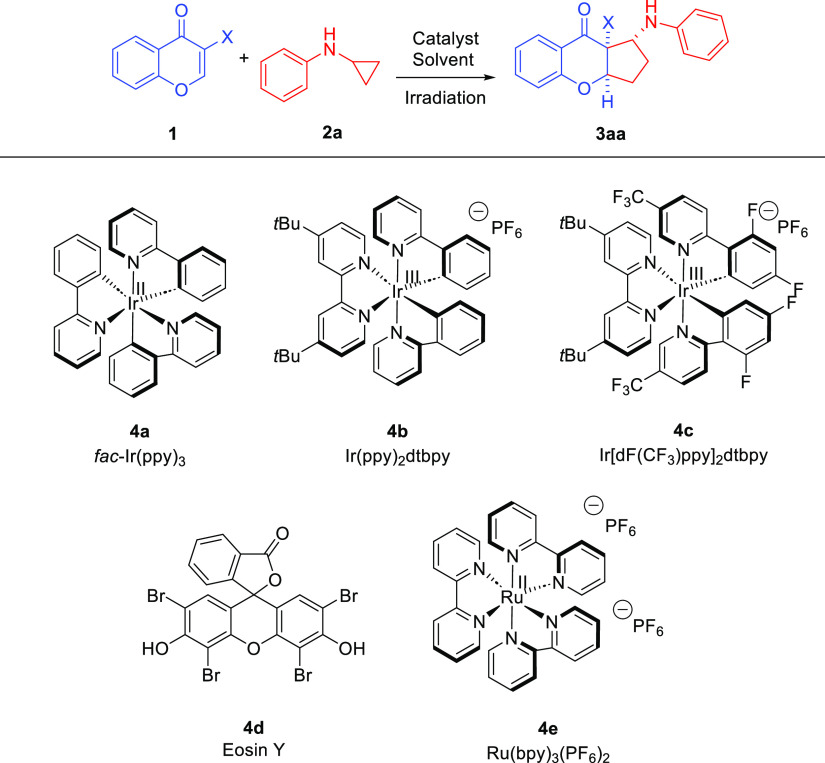
Visible-Light-Driven [2+3]-Photocycloaddition of 3-Cyanochromone **1** and *N*-Cyclopropyloaniline **2a**^a^

entry	catalyst/[mol%]	X	solvent	yield [%]	dr
1^b^	**4a/**5	H (**5a**)	CH_2_Cl_2_		
2^b^	**4a/**5	COOH (**5b**)	CH_2_Cl_2_		
3^b^	**4a/**5	C(O)Me (**5c**)	CH_2_Cl_2_		
4^b^	**4a/**5	CN (**1a**)	CH_2_Cl_2_	57	4.5:1
5^b^	**4b/**5	CN (**1a**)	CH_2_Cl_2_	41	5:1
6^b^	**4c/**5	CN (**1a**)	CH_2_Cl_2_	47	4.5:1
7^c^	**4d/**5	CN (**1a**)	CH_2_Cl_2_	72	4.5:1
8^b^	**4e/**5	CN (**1a**)	CH_2_Cl_2_	44	4.5:1
9^c^	**4d/**5	CN (**1a**)	CHCl_3_	47	5:1
10^c^	**4d/**5	CN (**1a**)	CCl_4_		
11^c^	**4d/**5	CN (**1a**)	MeOH	28	5:1
12^c^	**4d/**5	CN (**1a**)	MeCN	76	4.5:1
13^c^	**4d/**5	CN (**1a**)	DMSO	82	5:1
14^c^	**4d/**10	CN (**1a**)	DMSO	78	5:1
15^c^	**4d/**3	CN (**1a**)	DMSO	74	5:1
16^c^^,^^d^	**4d/**5	CN (**1a**)	DMSO	46	4:1
17^c^^,^^e^	**4d/**5	CN (**1a**)	DMSO	72	5:1
18^c^^,^^f^	**4d/**5	CN (**1a**)	DMSO	81	5:1
19^c^^,^^g^	**4d**/5	CN (**1a**)	DMSO	75	5:1
20		CN (**1a**)	DMSO		
21^h^	**4d**/5	CN (**1a**)	DMSO		
22^c^^,^^i^	**4d**/5	CN (**1a**)	DMSO		

a All reactions were performed at a 0.1 mmol scale using **1** (1.0 equiv) and **2a** (2.0 equiv) in the presence of the corresponding photoredox catalyst **4** (5 mol%) in the solvent (2 mL) for 24 h.

b Reaction performed under irradiation with the blue light (λ = 456 nm).

c Reaction performed under irradiation with the green light (λ = 525 nm).

d Reaction performed in DMSO (3 mL).

e Reaction performed in DMSO (1 mL).

f Reaction performed for 48 h.

g Reaction performed at a 1.0 mmol scale.

h Reaction performed in the dark.

i Reaction performed in the presence of TEMPO (1 equiv).

**Scheme 3:**
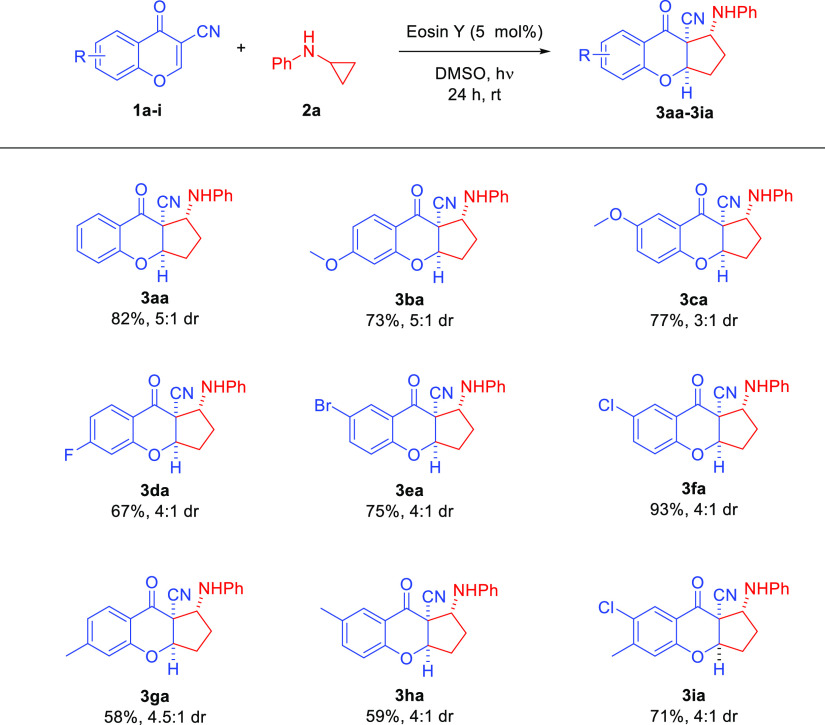
Photocatalytic [3+2]-Cycloaddition Initiated by Visible Light—Scope of Cyanochromones **1**

**Scheme 4:**
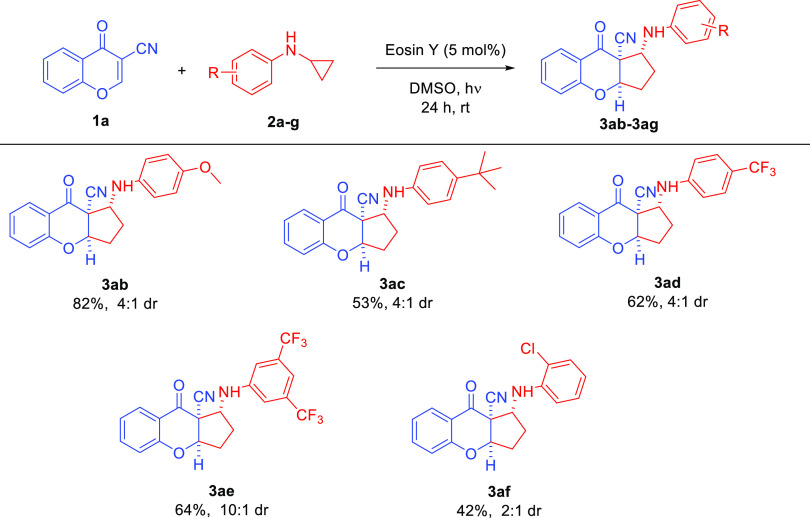
Photocatalytic [3+2]-Cycloaddition Initiated by Visible Light—Scope of *N*-Cyclopropyloanilines **2**

With the optimized reaction conditions in hand (Table 1, entry 13), the applicability of the developed methodology with regard to both reaction partners was examined (Schemes 3 and 4). Initially, various 3-cyanochromones **1a–i** containing either electron-withdrawing or donating substituents were tested in the [3+2]-photocycloaddition with *N*-cyclopropylaniline **2a** (Scheme 3). To our delight, the reaction proceeded efficiently, and in most cases, the desired products were obtained with yields in the range of 70–80%. Only for the 3-cyanochromones **1g**, **h** with a methyl substituent in the 6- and 7-position, the yield of the reaction lowered to 58 and 59%, respectively. In this context, it is worth noting that for the example with a chlorine substituent on the aromatic ring of 3-cyanochromone **1f** the cycloaddition proceeded with an excellent yield, and it was as high as 93%. Gratifyingly, the studies also indicated that the position of the substituent in 3-cyanochromone **1** had no pronounced influence on the reaction outcome, and the introduction of two substituents on the aromatic ring was also possible (Scheme 3, product **3ia**). In terms of diastereoselectivity, the cycloaddition was found to be unbiased toward the electronic properties of substituents, and it remained at a similar level to the model reaction.

In the second part of the scope studies, the possibility of employing various *N*-arylcyclopropylamines **2a–f** in the devised strategy was tested (Scheme 4). Unfortunately, it was found that the efficiency of the cascade reaction decreased, and target products **3ab**–**3af** were obtained in yields within the range of 42–82%. The lowest efficiency was obtained when *N*-cyclopropylaniline with an electron-accepting chlorine substituent was applied; in addition, the diastereoselectivity of the process decreased and amounted to only 2:1 dr (Scheme 4, product **3af**). In some of the cases, it was required to extend the reaction time from 24 to 72 h in order to achieve full conversion (Scheme 4, products **3ad**, **3ae**). To our delight, for the example with two trifluoromethyl groups on the aromatic ring of the *N*-cyclopropylaniline **2e** the diastereoselectivity of the cycloaddition increased to 10:1 dr. Unfortunately, for monosubstituted *N*-cyclopropylaniline **2f** the diastereomeric ratio remained at a moderate level.

**Scheme 5:**
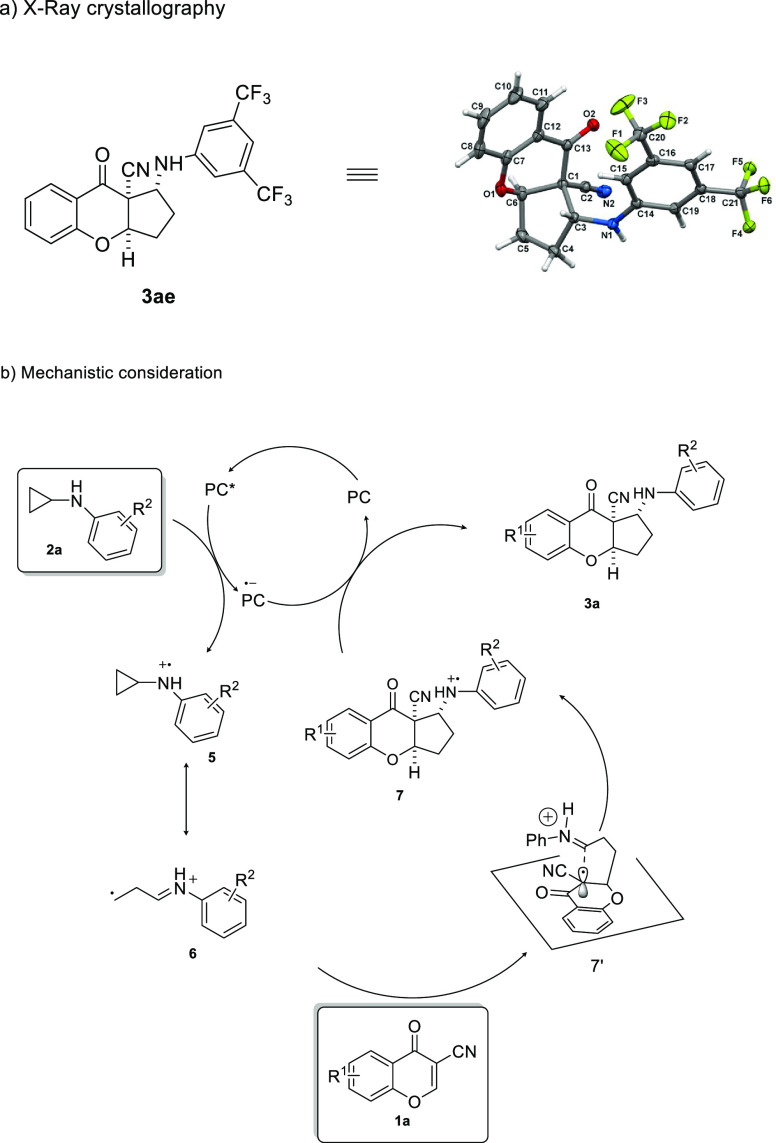
Photocatalytic [3+2]-Cycloaddition Initiated by Visible Light—Mechanistic Considerations and the X-ray Structure

In order to assign the relative configuration of target products **3**, single-crystal X-ray analysis was performed. To our delight, the major diastereoisomer of **3ae** provided crystals suitable for this experiment.^13^ The relative configuration of all major diastereoisomers of **3** was established by analogy (Scheme 5). To get insight into the reaction mechanism, a set of control experiments was performed (Table 1, entries 20–22) and the control reaction with TEMPO is consistent with a radical mechanism. Consequently, the catalytic cycle of the reaction and the stereochemical model accounting for the formation of the major diastereomer were proposed (Scheme 5b). It is postulated that the oxidation of *N*-cyclopropylaniline **2a** by a photoexcited catalyst initiates the reaction to form the corresponding amine radical cation **5**. Then ring-opening of **5** affords the distonic radical cation **6** and the cycloaddition leads to the annulation of the five-membered ring, yielding the amine radical **7**. The approach of radical **6** to substrate **1** is governed by steric factors. The subsequent reduction of **7** leads to the target product **3aa** and the regeneration of catalyst Eosin Y, which restarts the catalytic cycle.

**Scheme 6:**
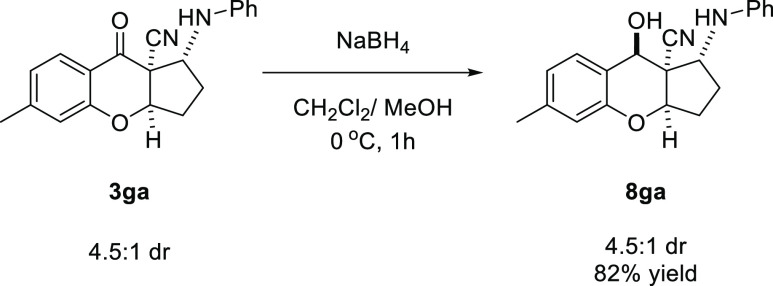
Chemo- and Diastereoselective Reduction of **3ga**

Subsequently, the product **3ga** was subjected to chemo- and diastereoselective reduction of the carbonyl group (Scheme 6). The reaction afforded alcohol **8ga** in 82% yield with a complete diastereoselectivity. The reaction occurred preferentially from the less hindered face of **3ga**.

## Conclusions

In summary, we have developed a new visible-light-mediated synthetic methodology, leading to the formation of hybrid molecules containing chromone and cyclopentane rings. The protocol was realized in the presence of 5 mol % of Eosin Y as a catalyst in dry DMSO as a solvent. The presented methodology gives access to fourteen functionalized cyclopenta[*b*]chromenocarbonitriles **3aa–3ag** under mild reaction conditions in good yields and diastereoselectivity. The utility of the obtained products was demonstrated in chemoselective reduction.

## Experimental Section

### General Methods

NMR spectra were acquired on a Bruker Ultra Shield 700 instrument, running at 700 MHz for ^1^H and at 176 MHz for ^13^C, respectively. Chemical shifts (δ) are reported in ppm relative to residual solvent signals (CDCl_3_: 7.26 ppm for ^1^H NMR, 77.16 ppm for ^13^C{^1^H} NMR. Mass spectra were recorded on a Bruker Maxis Impact time-of-flight mass spectrometry (ToF-MS) spectrometer using electrospray (ES+) ionization (referenced to the mass of the charged species). Analytical thin layer chromatography (TLC) was performed using precoated aluminum-backed plates (Merck Kieselgel 60 F254) and visualized by ultraviolet irradiation. Unless otherwise noted, analytical grade solvents and commercially available reagents were used without further purification. For flash chromatography (FC), silica gel (Silica gel, w/Ca, ∼0.1%), 230–400 mesh) was used. Green LED (50 W, λ = 525 nm) and blue LED (50 W, λ = 456 nm) were purchased from commercial supplier Kessil LED photoreactor lightning. Fluorescence measurements were performed using a Varian Cary Eclipse spectrofluorometer equipped with a thermos-stated cell holder. *N*-Cyclopropylanilines **2** were synthesized according to the literature procedure.^14^ 3-Cyanochromones **1** were prepared from the corresponding starting materials following the literature procedure.^15^

#### General Procedure for the Synthesis of Cyclopenta[*b*]chromene-9*a*-carbonitrile **3**

In a 10 mL Schlenk tube, 3-cyanochromone **1** (0.1 mmol, 1.0 equiv), *N*-cyclopropylaniline **2** (0.2 mmol, 2.0 equiv), and catalyst Eosin Y (5 mol%) were dissolved in dry DMSO (2 mL). The reaction mixture was degassed and filled three times with argon. Subsequently, the mixture was irradiated with a green LED for 24 h. Next, the reaction was quenched with water (5 mL), extracted with ethyl acetate (3 × 10 mL), and washed with brine (5 mL). The organic phase was dried over MgSO_4_ and concentrated under a reduced pressure. The crude product was purified by silica gel chromatography (*n*-hexane–ethyl acetate 10:1) to provide desired products **3**.

##### 9-Oxo-1-(phenylamino)-1,2,3,3*a*,9,9*a*-hexahydrocyclopenta[*b*]chromene-9*a*-carbonitrile (**3aa**)

The crude product was purified by silica gel chromatography (*n*-hexane:ethyl acetate 10:1) to provide desired product **3aa**. Pure product was isolated as yellow oil in 82% yield (24.9 mg), 5:1 dr, for the reaction performed at a 0.1 mmol scale, and 75% yield (228.2 mg), 5:1 dr, for the reaction performed at a 1.0 mmol scale. ^1^H NMR (700 MHz, CDCl_3_) δ 7.90 (ddd, *J* = 7.8, 1.8, 0.4 Hz, 1H, d_A_), 7.71–7.69 (m, 1H, d_B_), 7.59 (ddd, *J* = 8.4, 7.2, 1.8 Hz, 1H, d_A_), 7.53 (ddd, *J* = 8.0, 7.3, 1.8 Hz, 1H, d_B_), 7.12 (ddd, *J* = 8.1, 7.2, 1.0 Hz, 1H, d_A_), 7.10–7.06 (m, 4H, d_A+B_), 7.03–6.98 (m, 1H, d_A_, 2H, d_B_), 6.73–6.69 (m, 2H, d_A+B_), 6.52 (dt, *J* = 7.7, 1.1 Hz, 2H, d_A_), 6.44 (dt, *J* = 7.7, 1.1 Hz, 2H, d_B_), 5.16–5.14 (m, 2H, d_A+B_), 4.82 (q, *J* = 7.5, 7.0 Hz, 1H, d_B_), 4.72–4.67 (m, 1H, d_A_), 4.14 (d, *J* = 9.2 Hz, 1H, d_A_), 3.65 (d, *J* = 9.6 Hz 1H, d_B_), 2.81–2.73 (m, 1H, d_B_), 2.71 (dddd, *J* = 14.0, 9.5, 8.5, 5.6 Hz, 1H, d_A_), 2.57–2.48 (m, 2H, d_A+B_), 2.39–2.30 (m, 2H, d_A+B_), 2.07–1.99 (m, 1H, d_B_), 1.93 (dddd, *J* = 13.9, 11.9, 8.8, 5.2 Hz, 1H, d_A_). ^13^C{1H} NMR (176 MHz, CDCl_3_) δd_A_ 184.1, 159.0, 145.9, 137.6, 129.4 (2×C), 128.0, 123.0, 119.1, 118.4, 117.5, 115.3, 113.9 (2×C), 84.3, 59.7, 59.6, 30.1, 29.5. ^13^C{1H} NMR (176 MHz, CDCl_3_) δd_B_: 183.7, 159.7, 145.6, 137.1, 129.2, 127.6 (2×C), 122.9, 121.2, 120.5, 119.2, 118.1, 113.6 (2×C), 84.9, 64.2, 56.9, 32.8, 31.5. HRMS calculated for C_19_H_16_N_2_O_2_^+^ [M+H]^+^*m*/*z*: 305.1284, found: 305.1284.

##### 6-Methoxy-9-oxo-1-(phenylamino)-1,2,3,3*a*,9,9*a*-hexahydrocyclopenta[*b*]chromene-9*a*-carbonitrile (**3ba**)

The crude product was purified by silica gel chromatography (*n*-hexane:ethyl acetate 10:1) to provide the desired product **3ba**. Pure product was isolated as a yellow oil in 73% yield (23.7 mg), 5:1 dr. ^1^H NMR (700 MHz, CDCl_3_) δ 7.83 (d, *J* = 8.8 Hz, 1H, d_A_), 7.66 (d, *J* = 8.9 Hz, 1H, d_B_), 7.09 (tdd, *J* = 7.3, 3.9, 1.8 Hz, 4H, d_A+B_), 6.73–6.70 (m, 2H, d_A+B_), 6.67 (dd, *J* = 8.9, 2.4 Hz, 1H, d_A_), 6.57 (dd, *J* = 8.9, 2.4 Hz, 1H, d_B_), 6.55–6.53 (m, 2H, d_A_), 6.48 (dt, *J* = 7.6, 1.1 Hz, 2H, d_B_), 6.44 (dd, *J* = 3.5, 2.4 Hz, 2H, d_A+B_), 5.13–5.12 (m, 2H, d_A+B_), 4.80–4.73 (m, 1H, d_B_), 4.63 (q, *J* = 8.5 Hz, 1H, d_A_), 4.15 (d, *J* = 8.8 Hz, 1H, d_A_), 3.87 (s, 3H, d_A_), 3.86 (s, 3H, d_B_), 3.77 (d, *J* = 9.4 Hz, 1H, d_B_), 2.77–2.70 (m, 1H, d_B_), 2.68 (dddd, *J* = 13.8, 9.4, 8.4, 5.5 Hz, 1H, d_A_), 2.51 (ddt, *J* = 15.0, 11.8, 5.2 Hz, 1H, d_B_), 2.43 (ddt, *J* = 14.4, 9.0, 2.5 Hz, 1H, d_B_), 2.34 (dtd, *J* = 14.7, 9.8, 4.6 Hz, 1H, d_B_), 2.28 (dddd, *J* = 15.0, 9.5, 5.3, 1.5 Hz, 1H, d_A_), 2.03–1.95 (m, 1H, d_B_), 1.91 (dddd, *J* = 13.8, 11.7, 8.7, 5.4 Hz, 1H, d_A_). ^13^C{1H} NMR (176 MHz, CDCl_3_) δd_A_: 182.5, 167.3, 161.1, 146.1, 129.8, 129.4 (2×C), 119.0, 115.6, 113.9 (2×C), 111.6, 111.2, 101.3, 84.5, 59.7, 59.0, 56.0, 30.2, 29.5. ^13^C{1H} NMR (176 MHz, CDCl_3_) δd_B_ 182.1, 167.0, 161.7, 145.7, 129.5, 129.2 (2×C), 119.0, 117.7, 114.2, 113.6 (2×C), 111.1, 101.4, 84.9, 63.8, 56.2, 56.0, 32.5, 31.1. HRMS calculated for C_20_H_18_N_2_O_3_^+^ [M+H]^+^*m*/*z*: 335.1390, found: 335.1393.

##### 7-Methoxy-9-oxo-1-(phenylamino)-1,2,3,3*a*,9,9*a*-hexahydrocyclopenta[*b*]chromene-9*a*-carbonitrile (**3ca**)

The crude product was purified by silica gel chromatography (*n*-hexane:ethyl acetate 10:1) to provide desired product **3ca**. Pure product was isolated as pale brown oil in 77% yield (25.0 mg), 3:1 dr. ^1^H NMR (700 MHz, CDCl_3_) δ 7.28 (d, *J* = 3.2 Hz, 1H, d_A_), 7.19 (dd, *J* = 9.0, 3.2 Hz, 1H, d_A_), 7.12 (dd, *J* = 9.0, 3.2 Hz, 1H, d_B_), 7.11–7.07 (m, 5H, d_2A+3B_), 6.94 (d, *J* = 9.1 Hz, 2H, d_A+B_), 6.72 (tt, *J* = 7.4, 1.1 Hz, 2H, d_A+B_), 6.54–6.52 (m, 2H, d_A_), 6.47–6.43 (m, 2H, d_B_), 5.12 (dd, *J* = 4.8, 1.2 Hz, 1H, d_A_), 5.10 (dd, *J* = 4.3, 1.3 Hz, 1H, d_B_), 4.81 (t, *J* = 6.0 Hz, 1H, d_B_), 4.68 (t, *J* = 8.6 Hz, 1H, d_A_), 4.20–4.11 (m, 1H, d_A_), 4.10–4.08 (m, 1H, d_B_), 3.80 (s, 3H, d_A_), 3.71 (s, 3H, d_B_), 2.80–2.74 (m, 1H, d_B_), 2.70 (dddd, *J* = 13.9, 9.4, 8.4, 5.5 Hz, 1H, d_A_), 2.54–2.44 (m, 2H, d_A+B_), 2.34 (ddd, *J* = 15.3, 9.6, 4.5 Hz, 1H, d_B_), 2.29 (dddd, *J* = 14.9, 9.5, 5.2, 1.3 Hz, 1H, d_A_), 2.08–2.00 (m, 1H, d_B_), 1.92 (dddd, *J* = 13.8, 11.9, 8.8, 5.1 Hz, 1H, d_A_). ^13^C{1H} NMR (176 MHz, CDCl_3_) δd_A_: 184.2, 155.2, 153.6, 146.0, 129.4 (2×C), 127.0, 119.8, 119.1, 117.4, 115.4, 113.9 (2×C), 107.9, 84.5, 59.7, 59.6, 56.1, 30.2, 29.5. ^13^C{1H} NMR (176 MHz, CDCl_3_) δd_B_ 183.8, 155.1, 154.3, 145.6, 129.2 (2xC), 126.3, 120.5, 119.4, 119.2, 117.6, 113.7 (2×C), 107.8, 85.1, 64.2, 57.0, 56.0, 32.8, 31.4. HRMS calculated for C_20_H_18_N_2_O_3_^+^ [M+H]^+^*m*/*z*: 335.1390, found: 335.1393.

##### 6-Fluoro-9-oxo-1-(phenylamino)-1,2,3,3*a*,9,9*a*-hexahydrocyclopenta[*b*]chromene-9*a*-carbonitrile (**3da**)

The crude product was purified by silica gel chromatography (*n*-hexane:ethyl acetate 10:1) to provide the desired product **3da**. Pure product was isolated as a pale brown oil in 67% yield (21.0 mg), 4:1 dr. ^1^H NMR (700 MHz, CDCl_3_) δ 7.93 (dd, *J* = 8.8, 6.4 Hz, 1H, d_A_), 7.72 (dd, *J* = 8.8, 6.4 Hz, 1H, d_B_), 7.12–7.07 (m, 4H, d_A+B_), 6.85 (ddd, *J* = 8.8, 7.9, 2.4 Hz, 1H, d_A_), 6.75–6.69 (m, 2H, d_A_, 3H, d_B_), 6.54–6.51 (m, 2H, d_A_), 6.46 (dt, *J* = 7.6, 1.1 Hz, 2H, d_B_), 5.18–5.16 (m, 2H, d_A+B_), 4.81 (td, *J* = 8.6, 5.0 Hz, 1H, d_B_), 4.67 (q, *J* = 8.8 Hz, 1H, d_A_), 4.12 (d, *J* = 9.1 Hz, 1H, d_A_), 3.60 (d, *J* = 9.1 Hz, 1H, d_B_), 2.78 (dddd, *J* = 14.3, 10.2, 8.2, 2.8 Hz, 1H, d_B_), 2.70 (dddd, *J* = 13.9, 9.6, 8.5, 5.5 Hz, 1H, d_A_), 2.57–2.47 (m, 2H, d_A+B_), 2.37 (ddd, *J* = 14.7, 9.7, 4.5 Hz, 1H, d_B_), 2.31 (dddd, *J* = 15.1, 9.5, 5.3, 1.4 Hz, 1H, d_A_), 2.06–1.99 (m, 1H, d_B_), 1.93 (dddd, *J* = 14.0, 11.9, 8.8, 5.3 Hz, 1H, d_A_). ^13^C{1H} NMR (176 MHz, CDCl_3_) δd_A_ 182.7, 168.4 (d, *J* = 259.4 Hz), 160.8 (d, *J* = 13.5 Hz), 145.8, 130.7 (d, *J* = 11.5 Hz), 129.4 (2xC), 119.3, 115.0, 114.4 (d, *J* = 2.7 Hz), 113.9 (2×C), 111.7 (d, *J* = 22.8 Hz), 105.4 (d, *J* = 24.9 Hz), 84.9, 59.8, 59.4, 30.0, 29.4. ^13^C{1H} NMR (176 MHz, CDCl_3_) δd_B_ 182.4, 168.1 (d, *J* = 258.9 Hz), 161.4 (d, *J* = 13.5 Hz), 145.5, 130.3 (d, *J* = 11.5 Hz), 129.3 (2xC), 119.3, 117.3 (d, *J* = 2.5 Hz), 117.2, 113.5 (2×C), 111.3 (d, *J* = 22.5 Hz), 105.1 (d, *J* = 24.8 Hz), 85.4, 64.3, 56.8, 32.8, 31.5. HRMS calculated for C_19_H_15_FN_2_O_2_^+^ [M+H]^+^*m*/*z*: 323.1190, found: 323.1188.

##### 7-Bromo-9-oxo-1-(phenylamino)-1,2,3,3*a*,9,9*a*-hexahydrocyclopenta[*b*]chromene-9*a*-carbonitrile (**3ea**)

The crude product was purified by silica gel chromatography (*n*-hexane:ethyl acetate 10:1) to provide desired product **3ea**. Pure product was isolated as yellow oil in 75% yield (27.8 mg), 4:1 dr. ^1^H NMR (700 MHz, CDCl_3_) δ 7.99 (d, *J* = 2.5 Hz, 1H, d_A_), 7.79 (d, *J* = 2.5 Hz, 1H, d_B_), 7.67 (dd, *J* = 8.8, 2.5 Hz, 1H, d_A_), 7.60 (dd, *J* = 8.8, 2.5 Hz, 1H, d_B_), 7.13–7.09 (m, 4H, d_A+B_), 6.94–6.92 (m, 2H, d_A+B_), 6.76–6.73 (m, 2H, d_A+B_), 6.52 (dt, *J* = 7.7, 1.1 Hz, 2H, d_A_), 6.46 (dt, *J* = 7.6, 1.0 Hz, 2H, d_B_), 5.15 (dd, *J* = 4.9, 1.3 Hz, 1H, d_A_), 5.13 (dt, *J* = 4.3, 1.3 Hz, 1H, d_B_), 4.81 (td, *J* = 8.6, 4.9 Hz, 1H, d_B_), 4.68 (q, *J* = 8.9 Hz, 1H, d_A_), 4.11 (d, *J* = 9.4 Hz, 1H, d_A_), 3.56 (d, *J* = 9.0 Hz, 1H, d_B_), 2.78 (dddd, *J* = 14.2, 10.7, 8.2, 2.7 Hz, 1H, d_B_), 2.70 (dddd, *J* = 13.9, 9.5, 8.5, 5.5 Hz, 1H, d_A_), 2.53 (ddt, *J* = 15.0, 11.7, 5.2 Hz, 2H, d_A+B_), 2.38–2.34 (m, 1H, d_B_), 2.34–2.29 (m, 1H, d_A_), 2.03 (m, 1H, d_B_), 1.93 (dddd, *J* = 13.9, 11.8, 8.8, 5.2 Hz, 1H, d_A_). ^13^C{1H} NMR (176 MHz, CDCl_3_) δd_A_ 183.0, 157.9, 145.7, 140.3, 130.2, 129.5 (2×C), 120.5, 119.4, 118.8, 115.7, 114.9, 113.9 (2×C), 84.6, 59.9, 59.5, 30.0, 29.4. ^13^C{1H} NMR (176 MHz, CDCl_3_) δd_B_ 183.0, 158.6, 145.4, 139.6, 129.9, 129.3 (2×C), 121.6, 120.1, 119.5, 117.1, 115.6, 113.7 (2×C), 85.1, 64.8, 56.9, 32.9, 31.6. HRMS calcd for C_19_H_15_BrN_2_O_2_^+^ [M+H]^+^*m*/*z*: 383.0390, found: 383.0389.

##### 7-Chloro-9-oxo-1-(phenylamino)-1,2,3,3*a*,9,9*a*-hexahydrocyclopenta[*b*]chromene-9*a*-carbonitrile (**3fa**)

The crude product was purified by silica gel chromatography (*n*-hexane:ethyl acetate 10:1) to provide the desired product **3fa**. Pure product was isolated as a yellow oil in 93% yield (30.6 mg), 4:1 dr. ^1^H NMR (700 MHz, CDCl_3_) δ 7.84 (d, *J* = 2.6 Hz, 1H, d_A_), 7.64 (d, *J* = 2.6 Hz, 1H, d_B_), 7.53 (dd, *J* = 8.9, 2.6 Hz, 1H, d_A_), 7.46 (dd, *J* = 8.8, 2.6 Hz, 1H, d_B_), 7.13–7.08 (m, 4H, d_A+B_), 7.00–6.97 (m, 2H, d_A+B_), 6.76–6.73 (m, 2H, d_A+B_), 6.53–6.51 (m, 2H, d_A_), 6.48–6.43 (m, 2H, d_B_), 5.15 (d, *J* = 3.7 Hz, 1H, d_A_), 5.13 (d, *J* = 4.1 Hz, 1H, d_B_), 4.81 (td, *J* = 8.6, 4.9 Hz, 1H, d_B_), 4.68 (q, *J* = 8.8 Hz, 1H, d_A_), 4.12 (d, *J* = 9.4 Hz, 1H, d_A_), 3.57 (d, *J* = 9.1 Hz, 1H, d_B_), 2.81–2.76 (m, 1H, d_B_), 2.74–2.66 (m, 1H, d_A_), 2.57–2.47 (m, 2H, d_A+B_), 2.36 (ddd, *J* = 14.7, 9.7, 4.4 Hz, 1H, d_B_), 2.31 (ddd, *J* = 15.0, 9.5, 5.2 Hz, 1H, d_A_), 2.03 (dtt, *J* = 14.2, 9.2, 4.9 Hz, 1H, d_B_), 1.94 (dddd, *J* = 13.9, 11.9, 8.8, 5.2 Hz, 1H, d_A_). ^13^C{1H} NMR (176 MHz, CDCl_3_) δd_A_ 183.2, 157.4, 145.6, 137.5, 129.5 (2×C), 128.7, 127.1, 120.2, 119.4, 118.3, 114.9, 113.9 (2×C), 84.7, 59.9, 59.5, 30.0, 29.4. ^13^C{1H} NMR (176 MHz, CDCl_3_) δd_B_ 182.9, 158.2, 145.4, 136.9, 129.3 (2×C), 128.5, 126.8, 119.8, 119.5, 117.1, 115.3, 113.6 (2×C), 85.2, 64.7, 56.9, 32.9, 31.6. HRMS calculated for C_19_H_15_ClN_2_O_2_^+^ [M+H]^+^*m*/*z*: 339.0895, found: 339.0884.

##### 6-Methyl-9-oxo-1-(phenylamino)-1,2,3,3*a*,9,9*a*-hexahydrocyclopenta[*b*]chromene-9*a*-carbonitrile (**3ga**)

The crude product was purified by silica gel chromatography (*n*-hexane:ethyl acetate 10:1) to provide desired product **3ga**. Pure product was isolated as yellow oil in 58% yield (17.9 mg), 4.5:1 dr. ^1^H NMR (700 MHz, CDCl_3_) δ 7.78 (d, *J* = 8.0 Hz, 1H, d_A_), 7.60 (d, *J* = 8.0 Hz, 1H, d_B_), 7.11–7.07 (m, 4H, d_A+B_), 6.93 (ddd, *J* = 8.0, 1.5, 0.7 Hz, 1H, d_A_), 6.83 (ddd, *J* = 8.0, 1.6, 0.7 Hz, 1H, d_B_), 6.81 (s, 2H, d_A+B_), 6.74–6.68 (m, 2H, d_A+B_), 6.53 (dt, *J* = 7.7, 1.1 Hz, 2H, d_A_), 6.46 (dt, *J* = 7.6, 1.1 Hz, 2H, d_B_), 5.12 (td, *J* = 5.9, 5.4, 2.5 Hz, 2H, d_A+B_), 4.78 (td, *J* = 8.7, 4.9 Hz, 1H, d_B_), 4.66 (q, *J* = 8.2 Hz, 1H, d_A_), 4.14 (d, *J* = 8.0 Hz, 1H, d_A_), 3.72–3.69 (m, 1H, d_B_), 2.77–2.72 (m, 1H, d_B_), 2.69 (dddd, *J* = 13.9, 9.4, 8.5, 5.6 Hz, 1H, d_A_), 2.50 (ddt, *J* = 15.1, 11.9, 5.2 Hz, 1H, d_A_), 2.47–2.42 (m, 1H, d_B_), 2.40 (s, 3H, d_A_), 2.38 (s, 3H, d_B_), 2.36–2.32 (m, 1H, d_B_), 2.29 (dddd, *J* = 15.0, 9.5, 5.3, 1.4 Hz, 1H, d_A_), 2.00 (ddt, *J* = 14.3, 9.3, 4.8 Hz, 1H, d_B_), 1.91 (dddd, *J* = 13.8, 11.8, 8.7, 5.2 Hz, 1H, d_A_). ^13^C{1H} NMR (176 MHz, CDCl_3_) δd_A_ 183.6, 159.1, 149.5, 146.0, 129.4 (2×C), 127.9, 124.4, 119.0, 118.3, 115.5, 115.2, 113.9 (2×C), 84.3, 59.7, 59.4, 30.2, 29.5, 22.2. ^13^C{1H} NMR (176 MHz, CDCl_3_) δd_B_ 183.2, 159.8, 149.0, 145.7, 129.2 (2×C), 127.5, 124.2, 119.1, 118.2, 118.1, 117.6, 113.7 (2×C), 84.8, 64.0, 56.6, 32.7, 31.3, 22.2. HRMS calcd for C_20_H_18_N_2_O_2_^+^ [M+H]^+^*m*/*z*: 319.1441, found: 319.1445.

##### 7-Methyl-9-oxo-1-(phenylamino)-1,2,3,3*a*,9,9*a*-hexahydrocyclopenta[*b*]chromene-9*a*-carbonitrile (**3ha**)

The crude product was purified by silica gel chromatography (*n*-hexane:ethyl acetate 10:1) to provide the desired product **3ha**. Pure product was isolated as a pale brown oil in 59% yield (18.2 mg), 4:1 dr. ^1^H NMR (700 MHz, CDCl_3_) δ 7.68 (dd, *J* = 2.2, 1.0 Hz, 1H, d_A_), 7.50 (dd, *J* = 2.1, 1.0 Hz, 1H, d_B_), 7.39 (ddd, *J* = 8.5, 2.3, 0.7 Hz, 1H, d_A_), 7.33 (ddd, *J* = 8.4, 2.3, 0.7 Hz, 1H, d_B_), 7.16 (dd, *J* = 8.5, 7.4 Hz, 1H, d_B_), 7.11–7.07 (m, 4H, d_A+B_), 6.91 (dd, *J* = 8.4, 3.1 Hz, 1H, d_A_), 6.74–6.70 (m, 2H, d_A+B_), 6.53 (dt, *J* = 7.7, 1.1 Hz, 2H, d_A_), 6.46 (dt, *J* = 7.6, 1.1 Hz, 2H, d_B_), 5.12 (dd, *J* = 4.8, 1.2 Hz, 1H, d_A_), 5.11–5.10 (m, 1H, d_B_), 4.81–4.77 (m, 1H, d_B_), 4.67 (t, *J* = 8.6 Hz, 1H, d_A_), 4.20–4.10 (m, 1H, d_A_), 3.71–3.64 (m, 1H, d_B_), 2.78–2.73 (m, 1H, d_B_), 2.70 (dddd, *J* = 13.9, 9.5, 8.6, 5.6 Hz, 1H, d_A_), 2.51 (ddt, *J* = 15.1, 11.9, 5.2 Hz, 1H, d_A_), 2.47–2.41 (m, 1H, d_B_), 2.36–2.34 (m, 1H, d_B_), 2.33 (s, 3H, d_A_), 2.30 (dddd, *J* = 15.1, 9.5, 5.2, 1.3 Hz, 1H, d_A_), 2.25 (s, 3H, d_B_), 2.06–1.99 (m, 1H, d_B_), 1.92 (dddd, *J* = 13.9, 11.9, 8.7, 5.2 Hz, 1H, d_A_). ^13^C{1H} NMR (176 MHz, CDCl_3_) δd_A_ 184.2, 157.1, 146.0, 138.7, 132.7, 129.4 (2×C), 127.5, 119.1, 118.2, 117.1, 115.4, 113.9 (2×C), 84.3, 64.3, 59.6, 30.2, 29.5, 20.6. ^13^C{1H} NMR (176 MHz, CDCl_3_) δd_B_ 183.9, 157.8, 145.7, 138.1, 132.6, 129.2(2×C), 127.2, 120.1, 119.1, 117.9, 117.6, 113.7 (2×C), 84.9, 71.9, 56.9, 32.8, 31.4, 20.5. HRMS calculated for C_20_H_18_N_2_O_2_^+^ [M+H]^+^*m*/*z*: 319.1441, found: 319.1438.

##### 7-Chloro-6-methyl-9-oxo-1-(phenylamino)-1,2,3,3*a*,9,9*a*-hexahydrocyclopenta[*b*]chrom-ene-9*a*-carbonitrile (**3ia**)

The crude product was purified by silica gel chromatography (*n*-hexane:ethyl acetate 10:1) to provide desired product **3ia**. Pure product was isolated as yellow oil in 71% yield (24.4 mg), 4:1 dr. ^1^H NMR (700 MHz, CDCl_3_) δ 7.84 (s, 1H, d_A_), 7.65 (s, 1H, d_B_), 7.17–7.14 (m, 2H, d_B_), 7.10 (ddd, *J* = 8.6, 7.2, 2.2 Hz, 2H, d_A_), 6.92 (d, *J* = 0.9 Hz, 1H, d_A_), 6.91 (d, *J* = 0.9 Hz, 1H, d_B_), 6.73 (ddt, *J* = 8.3, 7.3, 1.1 Hz, 1H, d_A_), 6.69 (dt, *J* = 7.5, 1.1 Hz, 1H, d_B_), 6.52 (dt, *J* = 7.7, 1.0 Hz, 2H, d_A_), 6.47 (dt, *J* = 7.6, 1.1 Hz, 2H, d_B_), 5.12 (dd, *J* = 4.9, 1.3 Hz, 1H, d_A_), 5.11 (d, *J* = 4.2 Hz, 1H, d_B_), 4.78 (td, *J* = 8.7, 5.2 Hz, 1H, d_B_), 4.66 (q, *J* = 8.8 Hz, 1H, d_A_), 4.12 (d, *J* = 9.3 Hz, 1H, d_A_), 3.60 (d, *J* = 9.2 Hz, 1H, d_B_), 2.76 (dddd, *J* = 14.2, 10.2, 8.2, 2.7 Hz, 1H, d_B_), 2.68 (dddd, *J* = 13.9, 9.5, 8.5, 5.5 Hz, 1H, d_A_), 2.55–2.44 (m, 2H, d_A+B_), 2.42 (s, 3H, d_A_), 2.40 (s, 3H, d_B_), 2.37–2.32 (m, 1H, d_B_), 2.29 (dddd, *J* = 15.0, 9.5, 5.3, 1.4 Hz, 1H, d_A_), 2.00 (dtd, *J* = 14.3, 9.2, 5.1 Hz, 1H, d_B_), 1.92 (dddd, *J* = 13.9, 11.9, 8.8, 5.2 Hz, 1H, d_A_). ^13^C{1H} NMR (176 MHz, CDCl_3_) δd_A_ 182.9, 157.2, 147.1, 145.8, 129.4 (2×C), 129.4, 127.4, 120.4, 119.2, 116.4, 115.1, 113.9 (2×C), 84.6, 59.8, 59.4, 30.1, 29.4, 21.2. ^13^C{1H} NMR (176 MHz, CDCl_3_) δd_B_: 182.6, 158.0, 146.5, 145.6, 129.3 (2×C), 129.2, 127.1, 120.2, 119.4, 117.3, 115.2, 113.7 (2×C), 85.1, 64.6, 56.7, 32.9, 31.4, 21.1. HRMS calcd for C_20_H_17_ClN_2_O_2_^+^ [M+H]^+^*m*/*z*: 353.1051, found: 353.1052.

##### 1-((4-Methoxyphenyl)amino)-9-oxo-1,2,3,3*a*,9,9*a*-hexahydrocyclopenta[*b*]chromene-9*a*-carbonitrile (**3ab**)

The crude product was purified by silica gel chromatography (*n*-hexane:ethyl acetate 10:1) to provide the desired product **3ab**. Pure product was isolated as a pale brown oil in 82% yield (26.7 mg), 4:1 dr. ^1^H NMR (700 MHz, CDCl_3_) δ 7.88 (dd, *J* = 7.9, 1.6 Hz, 1H, d_A_), 7.72 (dd, *J* = 7.9, 1.6 Hz, 1H, d_B_), 7.60–7.56 (m, 1H, d_A_), 7.55–7.51 (m, 1H, d_B_), 7.13–7.09 (m, 2H, d_A+B_), 7.04–6.99 (m, 2H, d_A+B_), 6.70–6.65 (m, 4H, d_A+B_), 6.51–6.49 (m, 2H, d_A_), 6.41–6.39 (m, 2H, d_B_), 5.15 (d, *J* = 3.8 Hz, 1H, d_A_), 5.13 (d, *J* = 4.2 Hz, 1H, d_B_), 4.74–4.70 (m, 1H, d_B_), 4.63–4.58 (m, *J* = 6.4 Hz, 1H, d_A_), 3.95 (s, 3H, d_B_), 3.71 (s, 3H, d_A_), 2.77–2.72 (m, 1H, d_B_), 2.71–2.65 (m, 1H, d_A_), 2.53–2.45 (m, 2H, d_A+B_), 2.37–2.33 (m, 1H, d_B_), 2.33–2.28 (m, 1H, d_A_), 2.02 (dd, *J* = 9.4, 4.8 Hz, 1H, d_B_), 1.92 (dddd, *J* = 13.8, 12.0, 8.8, 5.2 Hz, 1H, d_A_). ^13^C{1H} NMR (176 MHz, CDCl_3_) δd_A_ 184.2, 159.0, 153.3, 139.9, 137.5, 128.0, 123.0, 118.4, 117.5, 115.7 (2×C), 115.4, 114.9 (2×C), 84.5, 61.1, 59.9, 55.8, 30.2, 29.5. ^13^C{1H} NMR (176 MHz, CDCl_3_) δd_B_ 183.9, 159.8, 153.2, 139.7, 137.0, 127.6, 122.9, 121.5, 118.1, 117.6, 115.1 (2×C), 114.7 (2×C), 84.9, 65.6, 57.0, 55.7, 32.7, 31.5. HRMS calculated for C_20_H_18_N_2_O_3_^+^ [M+H]^+^*m*/*z*: 335.1390, found: 335.1391.

##### 1-((4-(*tert*-Butyl)phenyl)amino)-9-oxo-1,2,3,3*a*,9,9*a*-hexahydrocyclopenta[*b*]chromene-9*a*-carbonitrile (**3ac**)

The crude product was purified by silica gel chromatography (*n*-hexane:ethyl acetate 10:1) to provide desired product **3ac**. Pure product was isolated as pale brown yellow oil in 53% yield (18.7 mg), 4:1 dr. ^1^H NMR (700 MHz, CDCl_3_) δ 7.91 (dd, *J* = 7.9, 1.6 Hz, 1H, d_A_), 7.72 (dd, *J* = 8.0, 1.6 Hz, 1H, d_B_), 7.59 (ddd, *J* = 8.5, 7.2, 1.8 Hz, 1H, d_A_), 7.52 (ddd, *J* = 8.4, 7.2, 1.8 Hz, 1H, d_B_), 7.21–7.15 (m, 1H, d_A_), 7.14–7.10 (m, 2H, d_A_, 4H, d_B_), 7.03–6.99 (m, 2H, d_A+B_), 6.66–6.63 (m, 1H, d_A_), 6.48–6.46 (m, 2H, d_A_), 6.44–6.41 (m, 2H, d_B_), 5.16–5.15 (m, 1H, d_A_), 5.14 (d, *J* = 4.3 Hz, 1H, d_B_), 4.77 (d, *J* = 4.8 Hz, 1H, d_B_), 4.63 (t, *J* = 8.1 Hz, 1H, d_A_), 4.07 (s, 1H, d_A_), 3.56 (d, *J* = 7.9 Hz, 1H, d_B_), 2.77**–**2.66 (m, 2H, d_A+B_), 2.55–2.45 (m, 2H, d_A+B_), 2.37–2.28 (m, 2H, d_A+B_), 2.06–1.99 (m, 1H, d_B_), 1.92 (dddd, *J* = 13.8, 11.9, 8.7, 5.2 Hz, 1H, d_A_), 1.24 (s, 3H), 1.23 (s, 9H). ^13^C{1H} NMR (176 MHz, CDCl_3_) δd_A_ 184.1, 159.0, 143.6, 141.9, 137.6, 128.0, 126.2 (2×C), 123.0, 118.4, 115.4, 115.1, 113.6 (2×C), 84.3, 60.1, 59.6, 34.0, 31.6 (3×C), 30.3, 29.6. ^13^C{1H} NMR (176 MHz, CDCl_3_) δd_B_: 183.8, 159.8, 143.9, 142.0, 137.0, 127.6, 126.0 (2×C), 122.9, 118.1, 117.6, 117.5, 113.5 (2×C), 84.9, 65.1, 57.0, 34.1, 32.9, 31.7 (3×C), 31.5. HRMS calcd for C_23_H_24_N_2_O_2_^+^ [M+H]^+^*m*/*z*: 361.1910, found: 361.1913.

##### 9-Oxo-1-((4-(trifluoromethyl)phenyl)amino)-1,2,3,3*a*,9,9*a*-hexahydrocyclopenta[*b*]chr-omene-9*a*-carbonitrile (**3ad**)

The crude product was purified by silica gel chromatography (*n*-hexane:ethyl acetate 10:1) to provide the desired product **3ad**. Pure product was isolated as a yellow oil in 62% yield (22.5 mg), 4:1 dr. ^1^H NMR (700 MHz, CDCl_3_) δ 7.91 (dd, *J* = 7.9, 1.7 Hz, 1H, d_A_), 7.69 (dd, *J* = 7.8, 1.7 Hz, 1H, d_B_), 7.61 (ddd, *J* = 8.7, 7.2, 1.7 Hz, 1H, d_A_), 7.55 (ddd, *J* = 8.7, 7.3, 1.8 Hz, 1H, d_B_), 7.31 (dd, *J* = 8.4, 6.1 Hz, 4H, d_A+B_), 7.17–7.13 (m, 1H, d_A_), 7.05–7.00 (m, 3H, d_A+2B_), 6.54 (d, *J* = 8.4 Hz, 2H, d_A_), 6.47 (d, *J* = 8.4 Hz, 2H, d_B_), 5.17 (td, *J* = 6.9, 5.9, 2.5 Hz, 2H, d_A+B_), 4.82 (td, *J* = 8.8, 5.2 Hz, 1H, d_B_), 4.72 (q, *J* = 8.8 Hz, 1H, d_A_), 4.49 (d, *J* = 9.1 Hz, 1H, d_A_), 4.03 (d, *J* = 9.3 Hz, 1H, d_A_), 2.82–2.76 (m, 1H, d_B_), 2.71 (dtd, *J* = 14.1, 9.0, 5.4 Hz, 1H, d_A_), 2.57–2.48 (m, 2H, d_A+B_), 2.38 (td, *J* = 10.0, 4.6 Hz, 1H, d_B_), 2.33 (dddd, *J* = 15.0, 9.5, 5.4, 1.5 Hz, 1H, d_A_), 2.07–2.01 (m, 1H, d_B_), 1.94 (dddd, *J* = 14.0, 11.9, 8.8, 5.4 Hz, 1H, d_A_). ^13^C{1H} NMR (176 MHz, CDCl_3_) δd_A_ 183.93, 159.04, 148.52, 137.89, 128.00, 126.78 (q, *J* = 3.8 Hz, 2×C), 124.74 (q, *J* = 270.5 Hz), 123.2, 118.52, 118.17, 117.37, 115.10, 112.90 (2×C), 84.19 (d, *J* = 3.0 Hz), 59.31, 58.92, 29.92, 29.40. ^13^C{1H} NMR (176 MHz, CDCl_3_) δd_B_ 183.58, 159.66, 148.00, 137.41, 127.61, 126.6 (q, *J* = 3.8 Hz, 2×C), 124.0 (q, *J* = 293.8 Hz), 123.07, 120.77, 120.58, 120.20, 117.16, 112.65 (2×C), 84.7 (d, *J* = 2.6 Hz), 63.04, 56.60, 32.56, 31.32. HRMS calcd for C_20_H_15_F_3_N_2_O_2_^+^ [M+H]^+^*m*/*z*: 373.1158, found: 373.1166.

##### 1-((3,5-Bis(trifluoromethyl)phenyl)amino)-9-oxo-1,2,3,3*a*,9,9*a*-hexahydrocyclopenta[*b*]-chromene-9*a*-carbonitrile (**3ae**)

The crude product was purified by silica gel chromatography (*n*-hexane:ethyl acetate 10:1) to provide desired product **3ae**. Pure product was isolated as yellow solid in 64% yield (27.6 mg), 10:1 dr. ^1^H NMR (700 MHz, CDCl_3_) δ 7.91 (dd, *J* = 7.9, 1.7 Hz, 1H), 7.63 (ddd, *J* = 8.7, 7.2, 1.7 Hz, 1H), 7.19–7.13 (m, 2H), 7.04 (dd, *J* = 8.4, 1.0 Hz, 1H), 6.85 (d, *J* = 1.4 Hz, 2H), 5.20 (dd, *J* = 4.8, 1.4 Hz, 1H), 4.74 (q, *J* = 8.5 Hz, 1H), 4.71 (t, *J* = 7.5 Hz, 1H), 2.76–2.68 (m, 1H), 2.55 (ddt, *J* = 15.0, 11.8, 5.1 Hz, 1H), 2.37 (dddd, *J* = 15.0, 9.4, 5.3, 1.4 Hz, 1H), 1.99 (dddd, *J* = 13.9, 11.8, 8.6, 5.3 Hz, 1H). ^13^C{1H} NMR (176 MHz, CDCl_3_) δ 184.1, 159.0, 146.7, 138.1, 132.6 (q, *J* = 33.0 Hz, 2×C), 128.1, 123.4 (q, *J* = 272.9 Hz, 2×C), 123.3, 118.5, 117.1, 115.0, 112.9 (dt, *J* = 3.8 Hz, 2×C), 112.0 (dt, *J* = 4.0 Hz), 84.2, 59.4, 59.0, 29.7, 29.4. HRMS calcd for C_21_H_14_F_6_N_2_O_2_^+^ [M+H]^+^*m*/*z*: 441.1032, found: 441.1035.

##### 1-((2-Chlorophenyl)amino)-9-oxo-1,2,3,3*a*,9,9*a*-hexahydrocyclopenta[*b*]chromene-9*a*-carbonitrile (**3af**)

The crude product was purified by silica gel chromatography (*n*-hexane:ethyl acetate 10:1) to provide desired product **3af**. Pure product was isolated as pale yellow oil in 42% yield (13.8 mg), 2:1 dr. ^1^H NMR (700 MHz, CDCl_3_) δ 7.91 (dd, *J* = 7.9, 1.6 Hz, 1H, d_A_), 7.65 (dd, *J* = 7.9, 1.5 Hz, 1H, d_B_), 7.60 (ddd, *J* = 8.5, 7.2, 1.8 Hz, 1H, d_A_), 7.53 (ddd, *J* = 8.9, 5.4, 1.8 Hz, 1H, d_B_), 7.25 (dd, *J* = 7.9, 1.5 Hz, 1H, d_A_), 7.15–7.11 (m, H, d_A+B_), 7.04–6.99 (m, 1H, d_A_, 2H, d_B_), 6.98–6.94 (m, 1H, d_A_), 6.81 (d, *J* = 7.9 Hz, 1H, d_B_), 6.65 (td, *J* = 7.8, 1.4 Hz, 1H, d_A_), 6.61 (td, *J* = 7.7, 1.4 Hz, 1H, d_B_), 6.43 (dd, *J* = 8.2, 1.0 Hz, 1H, d_A_), 5.19 (dd, *J* = 4.9, 1.9 Hz, 1H, d_A_), 5.16 (d, *J* = 4.1 Hz, 1H, d_B_), 4.88 (td, *J* = 9.0, 4.5 Hz, 1H, d_B_), 4.82 (d, *J* = 8.9 Hz, 1H, d_A_), 4.73 (q, *J* = 8.6 Hz, 1H, d_A_), 4.41 (d, *J* = 9.7 Hz, 1H, d_B_), 2.82 (dddd, *J* = 14.3, 10.7, 8.2, 2.8 Hz, 1H, d_B_), 2.74–2.67 (m, 1H, d_A_), 2.58–2.51 (m, 2H, d_A+B_), 2.42–2.36 (m, 1H, d_B_), 2.31 (dddd, *J* = 15.0, 9.5, 5.5, 1.9 Hz, 1H, d_A_), 2.13 (dtd, *J* = 13.9, 9.2, 4.5 Hz, 1H, d_B_), 2.02–1.96 (m, 1H, d_A_). ^13^C{1H} NMR (176 MHz, CDCl_3_) δd_A_ 183.9, 159.0, 142.0, 137.7, 129.7, 128.0, 127.6, 123.1, 120.1, 119.1, 118.5, 117.5, 115.0, 112.3, 84.3, 59.3, 59.3, 30.2, 29.3. ^13^C{1H} NMR (176 MHz, CDCl_3_) δd_B_ 183.6, 159.9, 141.2, 137.3, 129.0, 127.9, 127.3, 123.0, 120.3, 119.2, 118.9, 118.2, 117.3, 112.5, 85.0, 62.7, 57.2, 32.7, 31.7. HRMS calcd for C_19_H_15_ClN_2_O_2_^+^ [M+H]^+^*m*/*z*: 339.0895, found: 339.0890.

#### General Procedure for the Synthesis of 9-Hydroxy-6-methyl-1-(phenylamino)-1,2,3,3*a*,9,9*a*-hexahydrocyclopenta[*b*]chromene-9*a*-carbonitrile **8ga**

In a 4 mL vial, 6-methyl-9-oxo-1-(phenylamino)-1,2,3,3a,9,9a-hexahydrocyclopenta[*b*]chrom-ene-9*a*-carbonitrile **3ga** (17.6 mg, 0.055 mmol, 1.0 equiv) was dissolved in CH_2_Cl_2_ (0.4 mL). At 0 °C MeOH (0.1 mL) and NaBH_4_ (6.24 mg, 0.165 mmol, 3 equiv) were added. Reaction mixture was stirred for 1 h. Next, the reaction was quenched with water (5 mL), extracted with ethyl acetate (3 × 10 mL) and washed with brine (5 mL). The organic phase was dried over MgSO_4_ and concentrated under reduced pressure. The crude product was purified by silica gel chromatography (*n*-hexane:ethyl acetate 10:1) to provide desired product **8ga**. Pure product **8ga** was isolated as a pale yellow oil in 82% yield (14.4 mg), 4.5:1 dr. ^1^H NMR (700 MHz, CDCl_3_) δ 7.40 (d, *J* = 7.8 Hz, 1H), 7.20–7.14 (m, 2H), 6.89 (ddd, *J* = 7.8, 1.7, 0.8 Hz, 1H), 6.85–6.79 (m, 1H), 6.69 (s, 1H), 6.68– 6.60 (m, 2H), 5.26 (s, 1H), 4.83 (dd, *J* = 5.2, 2.9 Hz, 1H), 4.20 (s, 1H), 2.50–2.41 (m, 2H), 2.33 (s, 3H), 2.03–1.96 (m, 1H), 1.69 (s, 1H). ^13^C{1H} NMR (176 MHz, CDCl_3_) δ: 151.5, 145.7, 140.3, 129.7 (2C), 127.0, 123.6 (2C), 120.4, 120.2, 119.6, 117.6, 115.3, 81.1, 68.6, 56.8, 52.3, 30.0, 29.9, 29.6, 21.4. HRMS calculated for [C_20_H_20_N_2_O_2_H^+^]: 321.1598; found: 321.1599.

## Data Availability

The data underlying this study are available in the published article and its online Supporting Information.

## Supporting Information Available

Photochemical reaction setup; copies of ^1^H and ^13^C{^1^H} NMR spectra; cyclic voltammetry; fluorescence quenching; X-ray crystallography; unsuccessful substrates; and copies of ^1^H NMR and ^13^C NMR (PDF)
