# Supplementary material for: Visible Light Promoted [3+2]-Cycloaddition for the Synthesis of Cyclopenta[b]chromenocarbonitrile Derivatives
Source: J Org Chem. 2023 Nov 21;88(23):16589–97. doi: 10.1021/acs.joc.3c02172 (PMC10696553; doi:10.1021/acs.joc.3c02172)

Supporting Information

Visible light promoted [3+2]-cycloaddition for the synthesis of cyclopenta[*b*]chromenocarbonitrile derivatives

Ewelina Kowalska,[a] Mateusz Dyguda,[a] Angelika Artelska[b] and Anna Albrecht [c*]

[a] Institute of Organic Chemistry, Lodz University of Technology, Żeromskiego 116, 90-924 Łódź, Poland

[b] Institute of Applied Radiation Chemistry, Lodz University of Technology, Żeromskiego 116, 90-924 Łódź, Poland

[c] Institute of General and Ecological Chemistry, Department of Chemistry, Lodz University of Technology, Żeromskiego 116, 90-924 Łódź, Poland

anna.albrecht@p.lodz.pl

Contents

1. General Methods……………………………………………………………..….S2
2. Cyclic voltammetry ……………………………………………………….......…S4
3. Fluorescence Quenching…………………………….……………………….….S5
4. X-Ray crystallography………………………………………………………S7
5. Unsuccessful substrates………………………………………………………….S8
6. Copies of ^{1}H NMR and ^{13}C NMR………………………………….…...…S9

1. General methods

NMR spectra were acquired on a Bruker Ultra Shield 700 instrument, running at 700 MHz for ^{1}H and 176 MHz for ^{13}C, respectively. Chemical shifts (δ) are reported in ppm relative to residual solvent signals (CDCl3: 7.26 ppm for 1H NMR, 77.16 ppm for 13C{1H} NMR. Mass spectra were recorded on a Bruker Maxis Impact Time-of-Flight Mass Spectrometer (ToF-MS) using electrospray (ES+) ionization (referenced to the mass of the charged species). Analytical thin layer chromatography (TLC) was performed using pre-coated aluminum-backed plates (Merck Kieselgel 60 F254) and visualized by ultraviolet irradiation. Unless otherwise noted, analytical grade solvents and commercially available reagents were used without further purification. For flash chromatography (FC) silica gel (Silica gel, w/Ca, ~0.1%), 230-400 mesh). Green LED (50 W, λ = 525 nm), blue LED (50 W, λ = 456 nm), were purchased from commercial supplier Kessil LED photoreactor lightning. Fluorescence measurements were performed using Varian Cary Eclipse spectrofluorometer equipped with thermos stated cell holder. N-cyclopropylanilines **2** were synthetized according to the literature procedure[1]. 3-Cyanochromones **1** were prepared from the corresponding starting materials following the literature procedure[2].

Figure S1 shows the 50W 525 nm photochemical reaction setup. The reaction vials in front of the 50W 525 nm bulb at approximatively 4.5 cm distance. To maintain a stable reaction temperature a fan were placed in close proximity to the reaction vials (23±2 °C).

[1] Nguyen, T. H., Morris, S. A., & Zheng, N. *Advanced synthesis & catalysis*, **2014**, *356*(13), 2831-2837.

[2] Reddy, G. J., Latha, D., Thirupathaiah, C., & Rao, K. S. *Tetrahedron letters*, **2004**, *45*(4), 847-848.

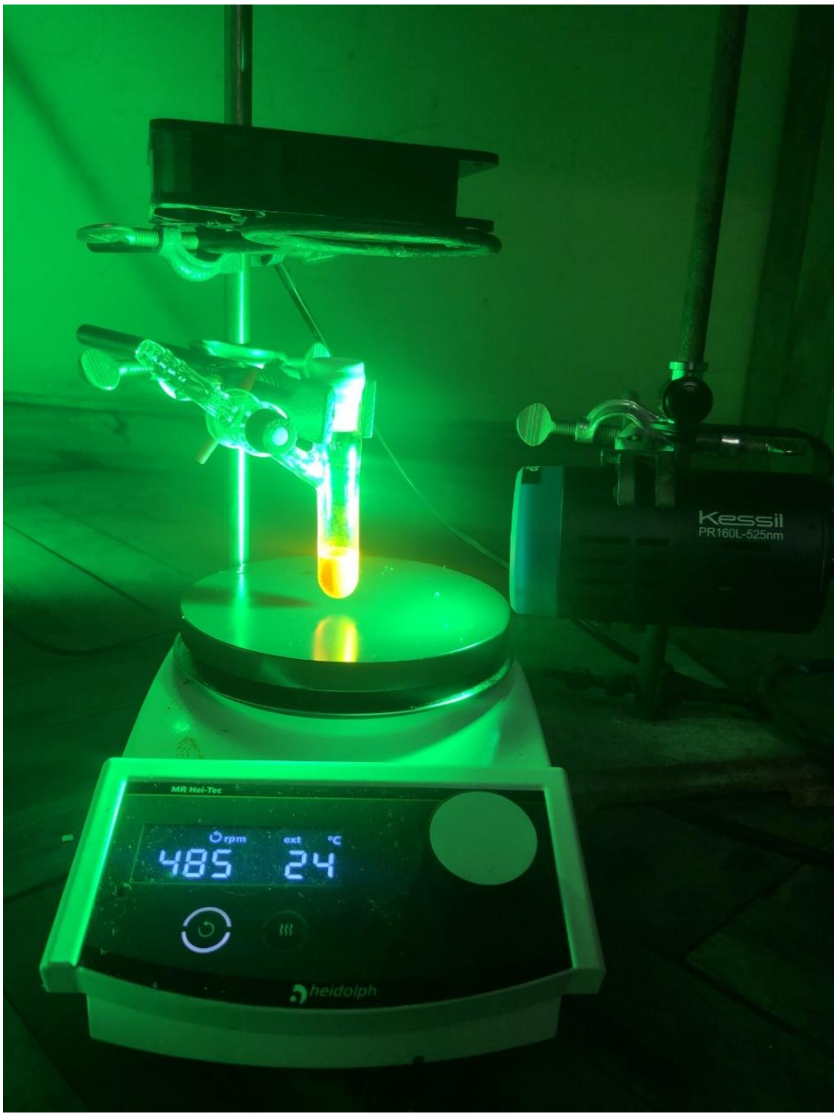

Fig. S1 Photochemical reaction setup using 50W 525nm Kessil LED.

2. Cyclic Voltammetry

Cyclic Voltammetry of *N*-cyclopropylaniline[3] **2a** and 3-cyanochromone[4] **1a** are described in the literature.

3. Fluorescence Quenching

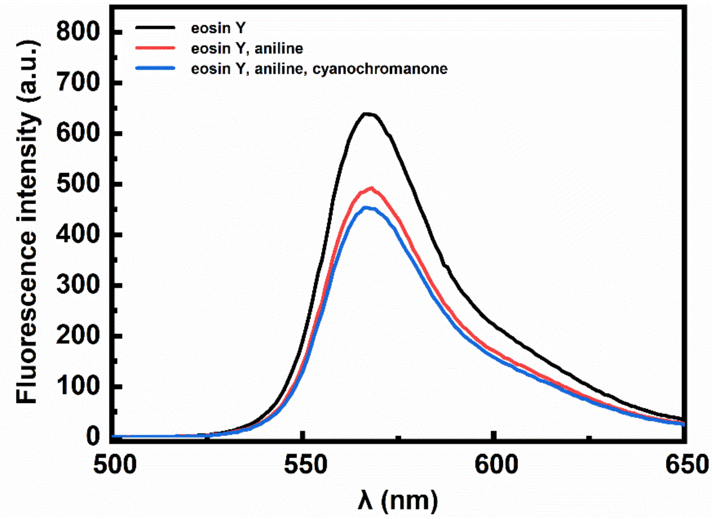

Fig. S2 Fluoresence spectra of Eosin Y (20 μM) in DMSO, Eosin Y (20 μM) and *N*-cyclopropylaniline (800 μM) in DMSO, Eosin Y (20 μM), *N*-cyclopropylaniline (800 μM) and 3-cyanochromone (400 μM) in DMSO. Excitation and emission wavelength were 324 nm and 568 nm.

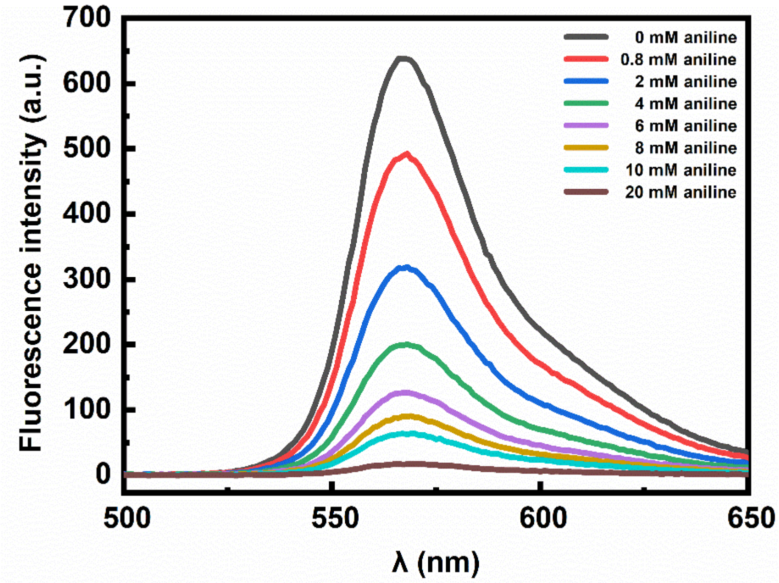

Fig. S3 Fluorescence quenching of Eosin Y (20 μM) by *N*-cyclopropylaniline **2a** in DMSO. Excitation and emission wavelength were 324 nm and 568 nm.

---

[3] Cai, Y., Wang, J., Zhang, Y., Li, Z., Hu, D., Zheng, N., & Chen, H. *Journal of the American Chemical Society*, **2017**, *139*(35), 12259-12266.

[4] Moczulski, M., Artelska, A., Albrecht, Ł., & Albrecht, A. *European Journal of Organic Chemistry*, **2022**, *2022*(31), e202200630.

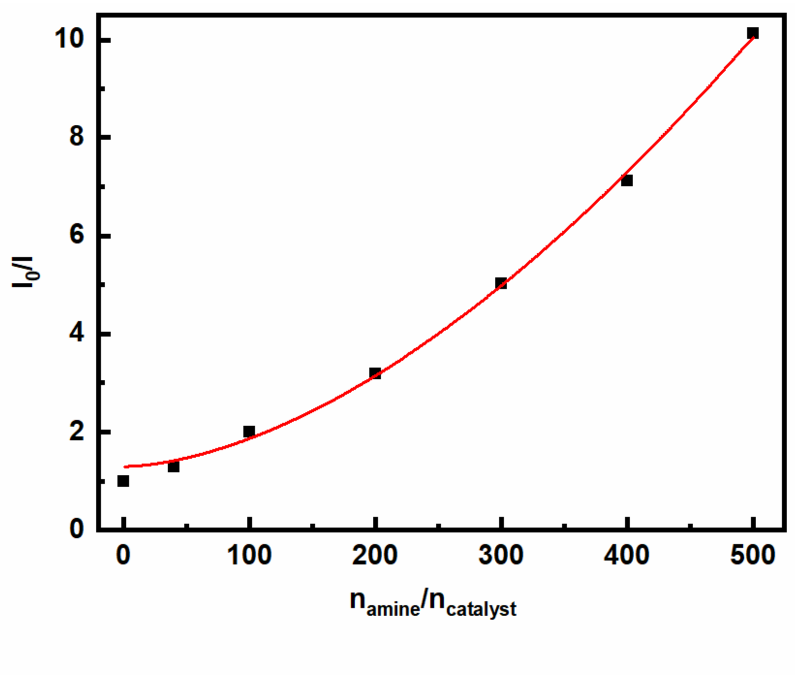

Fig. S4 Stern-Volmer plot of Fluorensence quenching of Eosin Y by *N*-cyclopropylaniline **2a**.

The fluorescence quenching of the catalyst (eosin Y) by aniline was studied. In the absence of aniline, the emission spectrum of eosin Y shows a maximum at 568 nm. Increasing the concentration of the quencher results in a decrease in the intensity of this emission. At a sufficiently high concentration of aniline (20 mM), the eosin Y fluorescence is almost completely extinguished and the new emission is symmetrical about the peak maximum. In all solutions, the emission spectrum of eosin Y is not significantly affected by aniline. Based on the measured emission spectra, a Stern-Volmer plot was prepared. A positive deviation of the Stern-Volmer plot (Fig. S4) from the expected linear relationship is observed. The linearity of the Stern-Volmer plot indicates that one type of extinction mechanism is dominant - dynamic (collision) extinction. Positive deviations from the linearity of the Stern-Volmer plots suggest that the quenching process proceeds simultaneously by two mechanisms, one of which is associated with dynamic quenching. The second mechanism is associated with static quenching by the formation of a non-fluorescent complex between the fluorophore and the quencher in the ground state or by the "instantaneous" absorption of energy by a very close (Van der Waals radius) quencher particle. Fluorescence quenching is an important method to study the energetics of excited states.

4. X-Ray crystallography

Crystal of compound **3ae**, was obtained as colourless needles via evaporation of a petroleum ether/dichloromethane solution. The crystal structure of the compound **3ae**, $C_{21}H_{14}F_6N_2O_2$, was established by single-crystal X-ray diffraction at 100 K. The compound crystallizes as a racemate in the centrosymmetric triclinic space group *P*-1 (Z = 4) and the crystal structure consists of the two enantiomer molecules of a racemic compound in the unit cell (Figure S5).

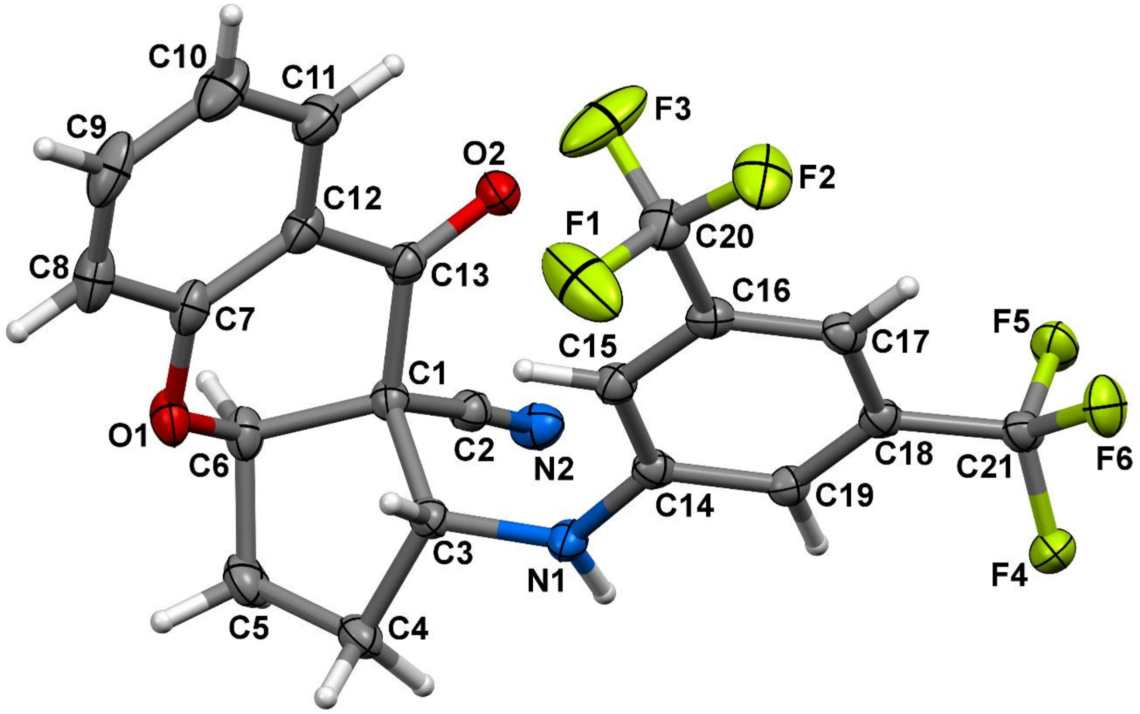

Figure S5. The molecular structure of the compound **3ae** (one of the racemic enantiomers) at 100 K, with the atom labeling scheme. Displacement ellipsoids are shown at the 50% probability level. Hydrogen atoms are drawn with an arbitrary radius.

Single crystal X-ray diffraction data were collected at 100 K by the ω-scan technique using a RIGAKU XtaLAB Synergy, Dualflex, Pilatus 300K diffractometer[5] with PhotonJet micro-focus X-ray Source Cu-Kα (λ = 1.54184 Å). Data collection, cell refinement, data reduction and absorption correction were performed using CrysAlis PRO software.[5] The crystal structure was solved by using direct methods with the SHELXT 2018/2 program.[6] Atomic scattering factors were taken from the International Tables for X-ray Crystallography. Positional parameters of

[5] Rigaku OD. CrysAlis PRO. Rigaku Oxford Diffraction Ltd, Yarnton, Oxfordshire, England, 2019.

[6] Sheldrick, G.M. "SHELXT - integrated space-group and crystal-structure determination", *Acta Cryst*. **2015**, A71, 3-8.

non-H-atoms were refined by a full-matrix least-squares method on F^2 with anisotropic thermal parameters by using the SHELXL-2019/2 program[7]. Fluorine atoms in one of the –CF3 groups have been found to be disordered and refined over three positions with equal occupancy sites of 1/3. All carbon hydrogens were positioned geometrically and refined by a riding model with U_{iso} 1.2 times that of attached atoms and remaining hydrogen atoms were found by Fourier difference and refined freely.

3ae: Formula $C_{21}H_{14}F_6N_2O_2$, triclinic, space group *P*-1, *Z* = 4, unit cell constants *a* = 8.1914(1), *b* = 15.2464(1), *c* = 15.6254(1) Å, α = 80.429(1), β = 87.313(1), γ = 76.928(1)°, *V* = 1874.33(3) $Å^3$. The integration of the data yielded a total of 69153 reflections with θ angles in the range of 3.82 to 74.48°, of which 7388 were unique (R_{int} = 1.71%). The final anisotropic full-matrix least-squares refinement on F^2 with 622 parameters. The final R_1 was 0.0326 (for $I > 2\sigma(I)$) and wR_2 was 0.0798 (all data). The largest peak in the final difference electron density synthesis was 0.408 $eÅ^{-3}$ and the largest hole was -0.479 $eÅ^{-3}$. The goodness-of-fit was 1.030.

CCDC 2268189 contains the supplementary crystallographic data for this paper. These data can be obtained free of charge from The Cambridge Crystallographic Data Centre via www.ccdc.cam.ac.uk/structures

[7] Sheldrick, G.M. "Crystal structure refinement with SHELXL", *Acta Cryst.* 2015, C**71**, 3-8.

5. Unsuccessful substrates

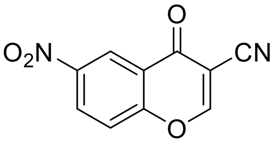

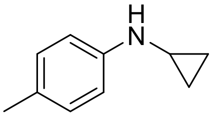

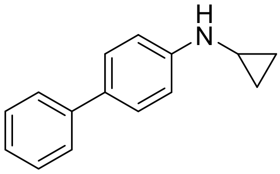

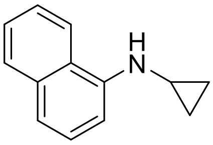

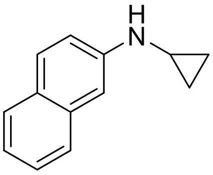

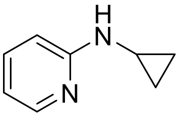

6. NMR Data

9-Oxo-1-(phenylamino)-1,2,3,3a,9,9a-hexahydrocyclopenta[*b*]chromene-9a-carbonitrile (*3aa*) ^{1}H NMR (700 MHz, $CDCl_3$)

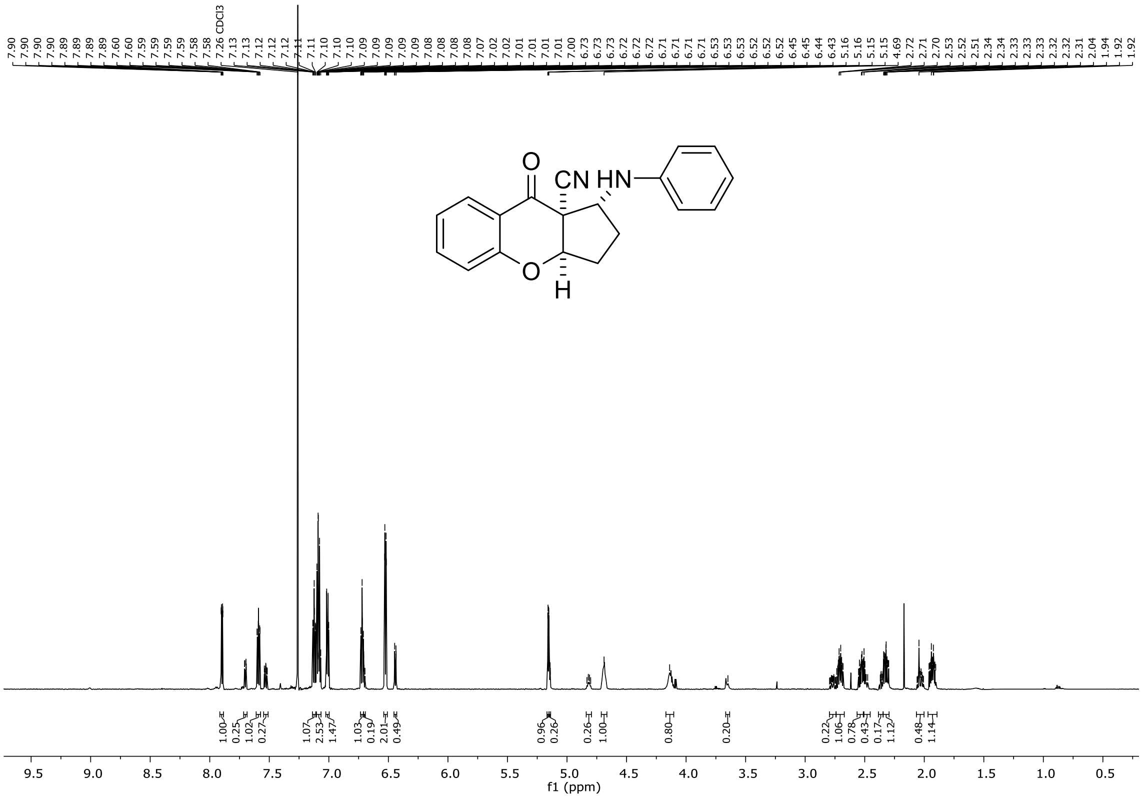

^{13}C{1H} NMR (176 MHz, $CDCl_3$)

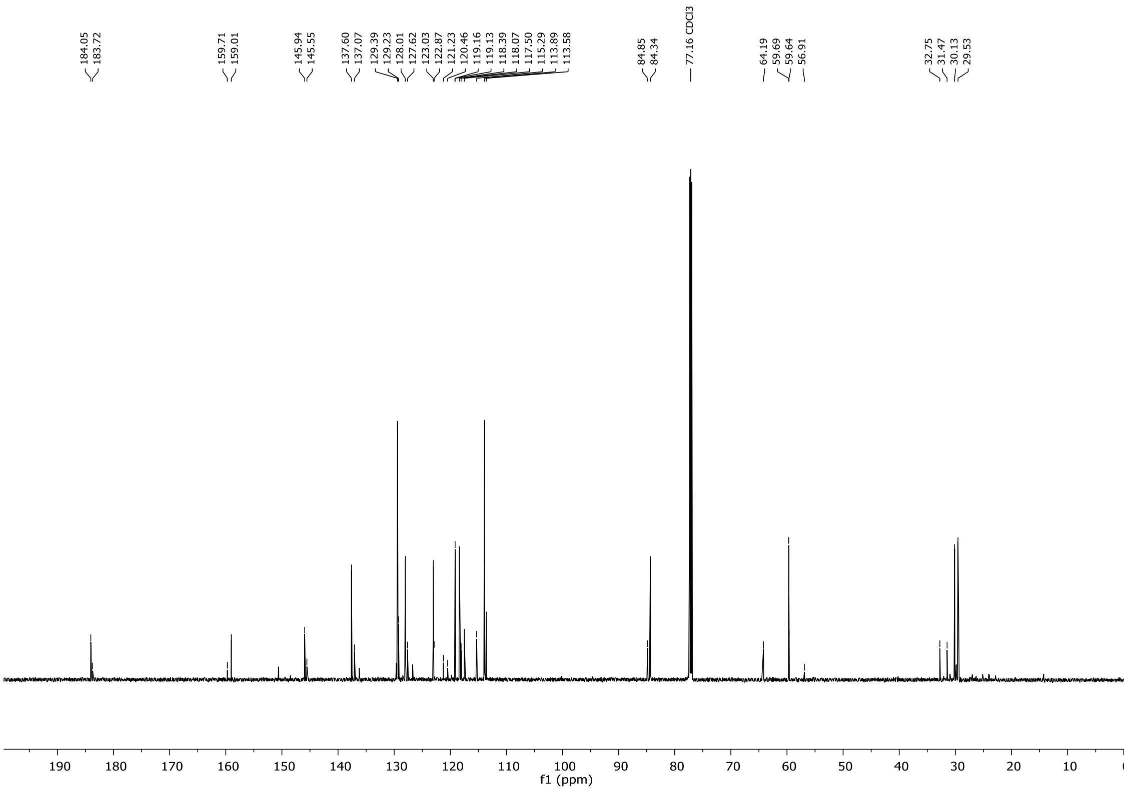

6-Methoxy-9-oxo-1-(phenylamino)-1,2,3,3a,9,9a-hexahydrocyclopenta[*b*]chromene-9a-carbonitrile (*3ba*)

^{1}H NMR (700 MHz, CDCl$_3$)

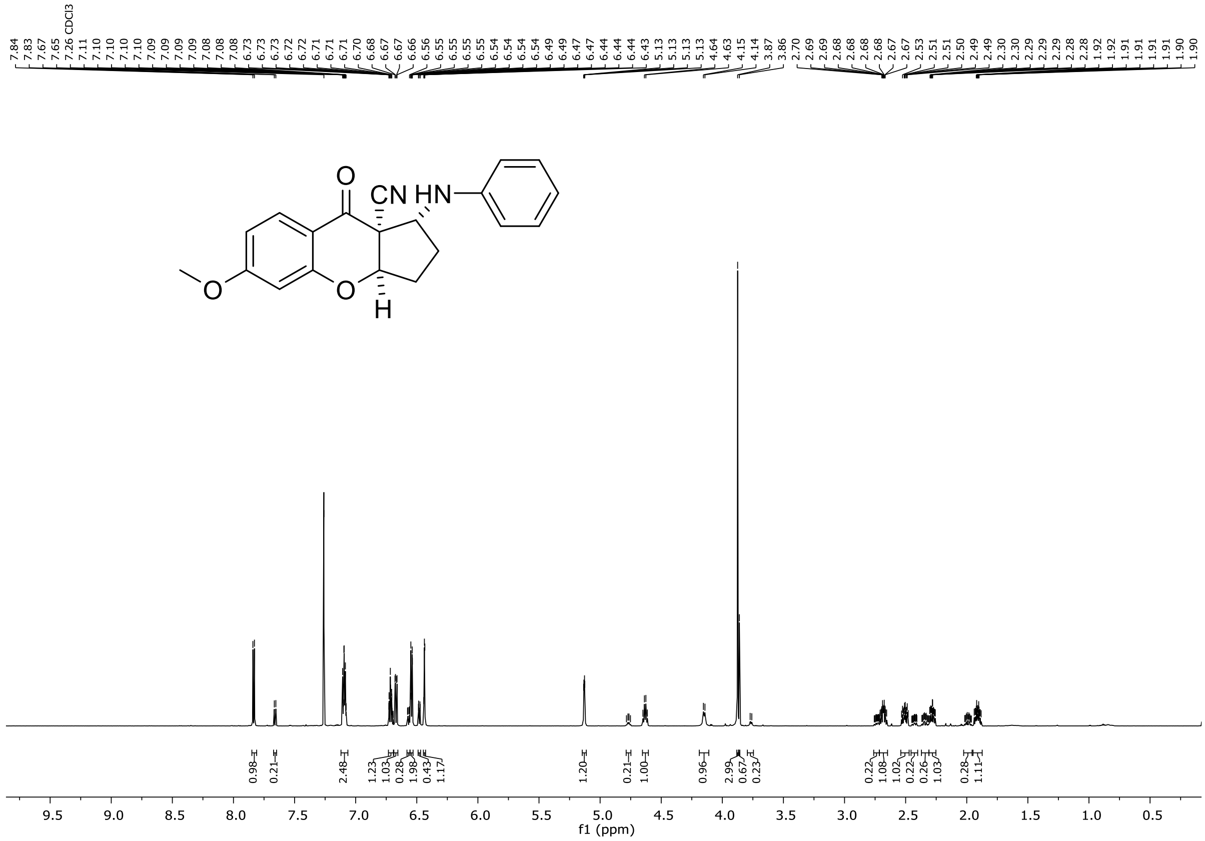

^{13}C{1H} NMR (176 MHz, CDCl$_3$)

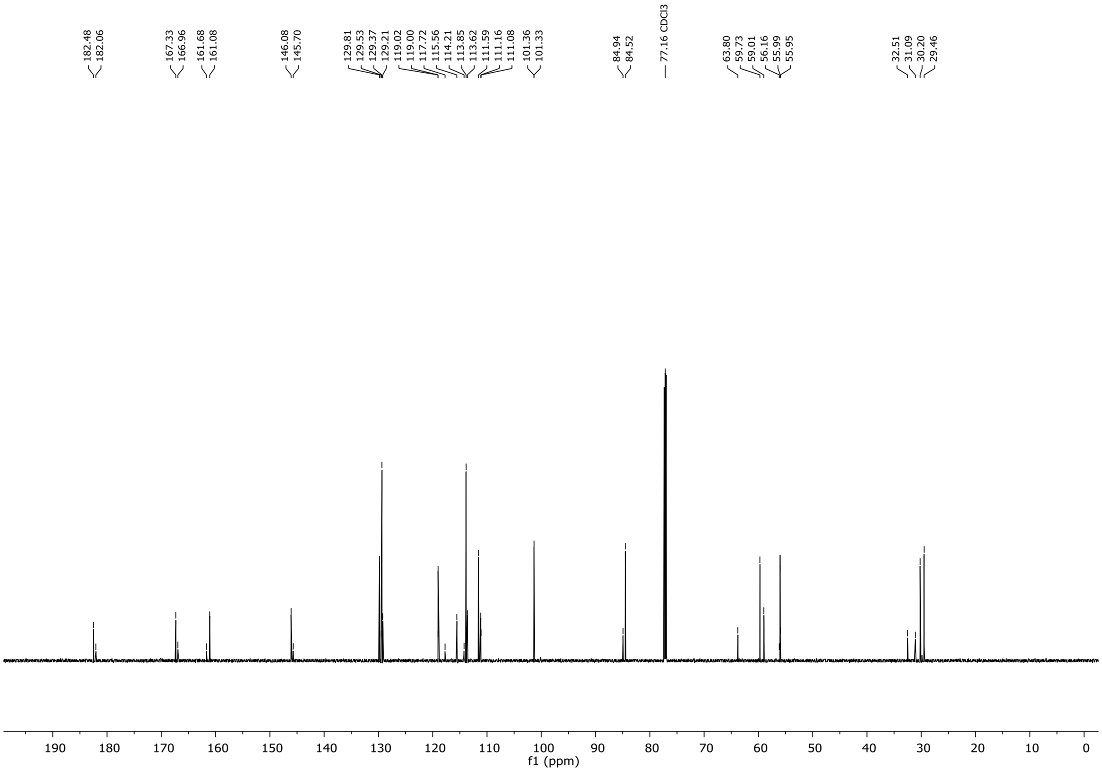

7-Methoxy-9-oxo-1-(phenylamino)-1,2,3,3a,9,9a-hexahydrocyclopenta[*b*]chromene-9a-carbonitrile (*3ca*)

^{1}H NMR (700 MHz, $CDCl_3$)

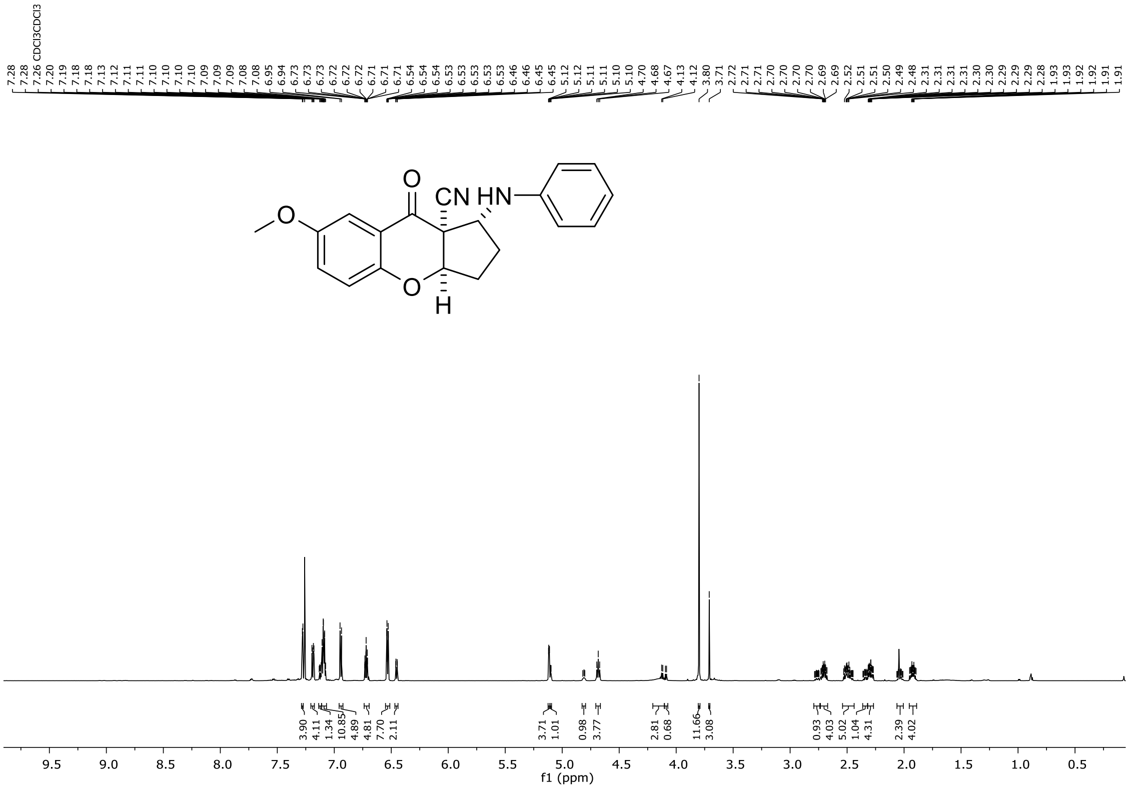

^{13}C{1H} NMR (176 MHz, $CDCl_3$)

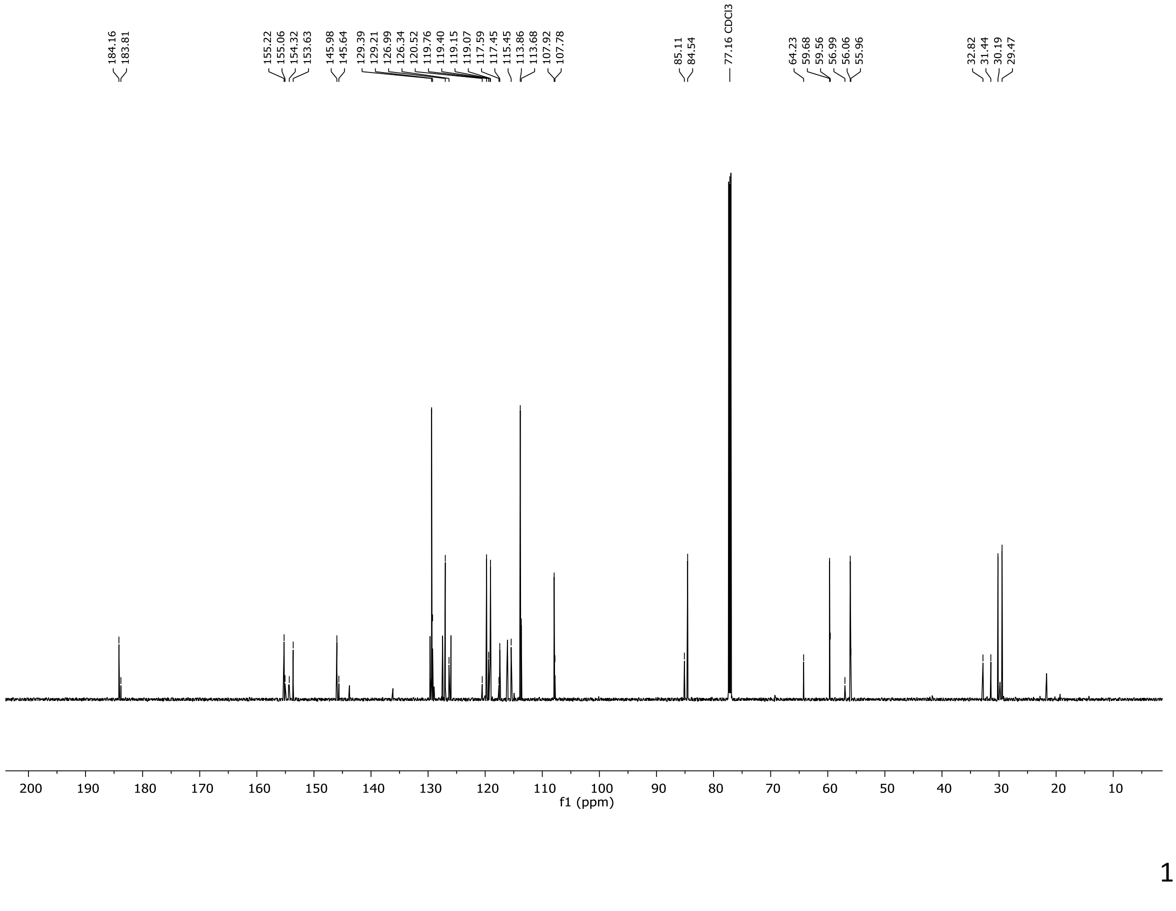

6-Fluoro-9-oxo-1-(phenylamino)-1,2,3,3a,9,9a-hexahydrocyclopenta[*b*]chromene-9a-carbonitrile (*3da*)

^{1}H NMR (700 MHz, $CDCl_3$)

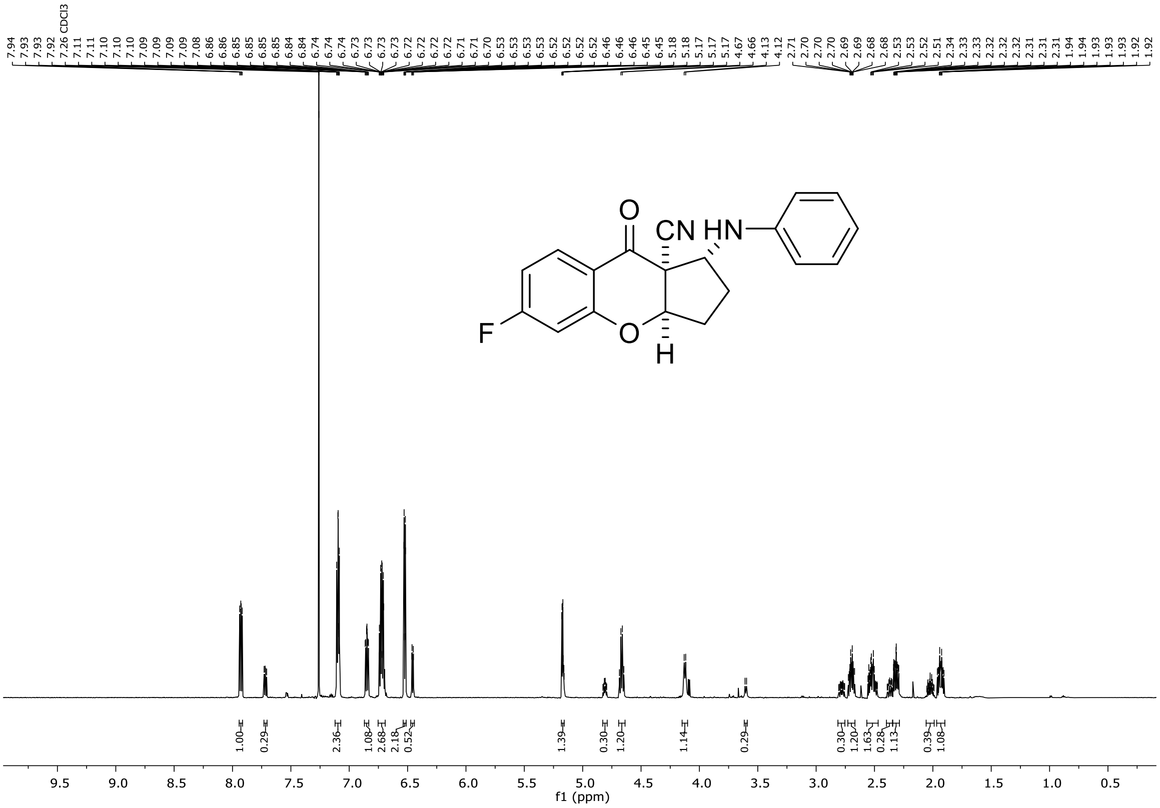

^{13}C{1H} NMR (176 MHz, $CDCl_3$)

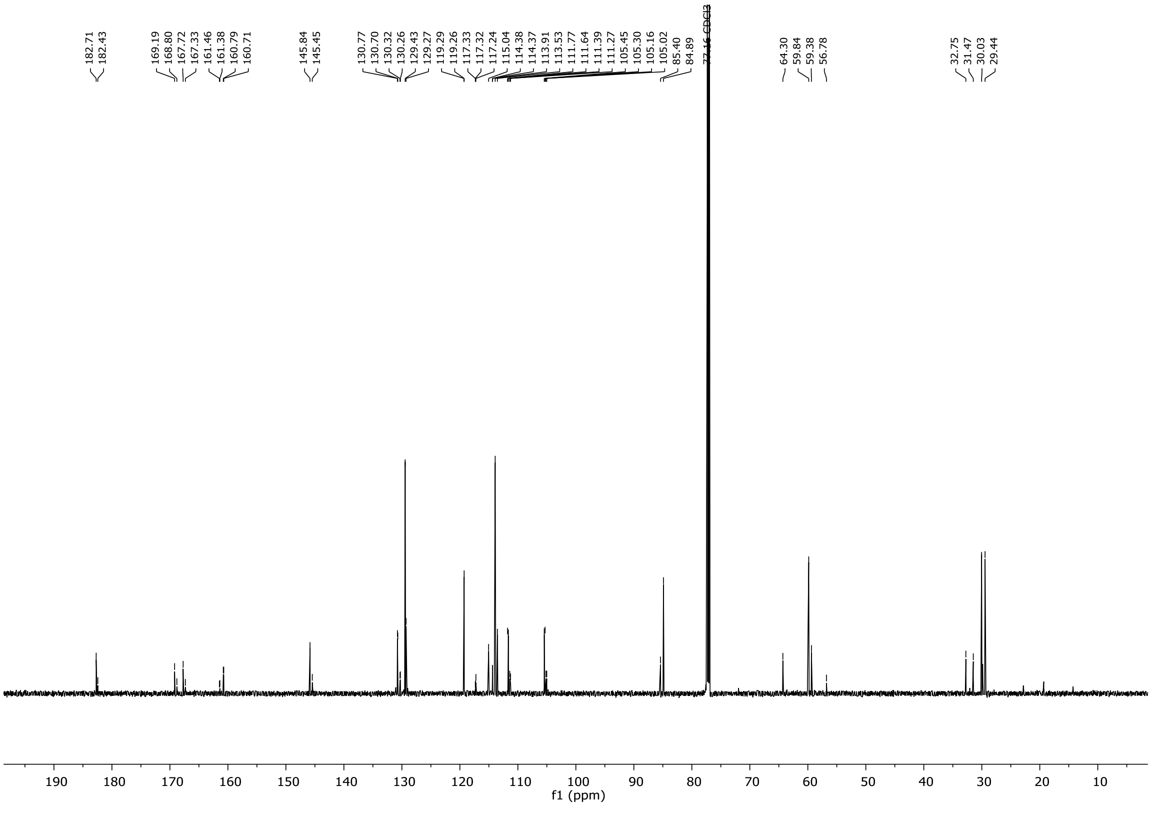

7-Bromo-9-oxo-1-(phenylamino)-1,2,3,3a,9,9a-hexahydrocyclopenta[*b*]chromene-9a-carbonitrile (*3ea*)

^{1}H NMR (700 MHz, $CDCl_3$)

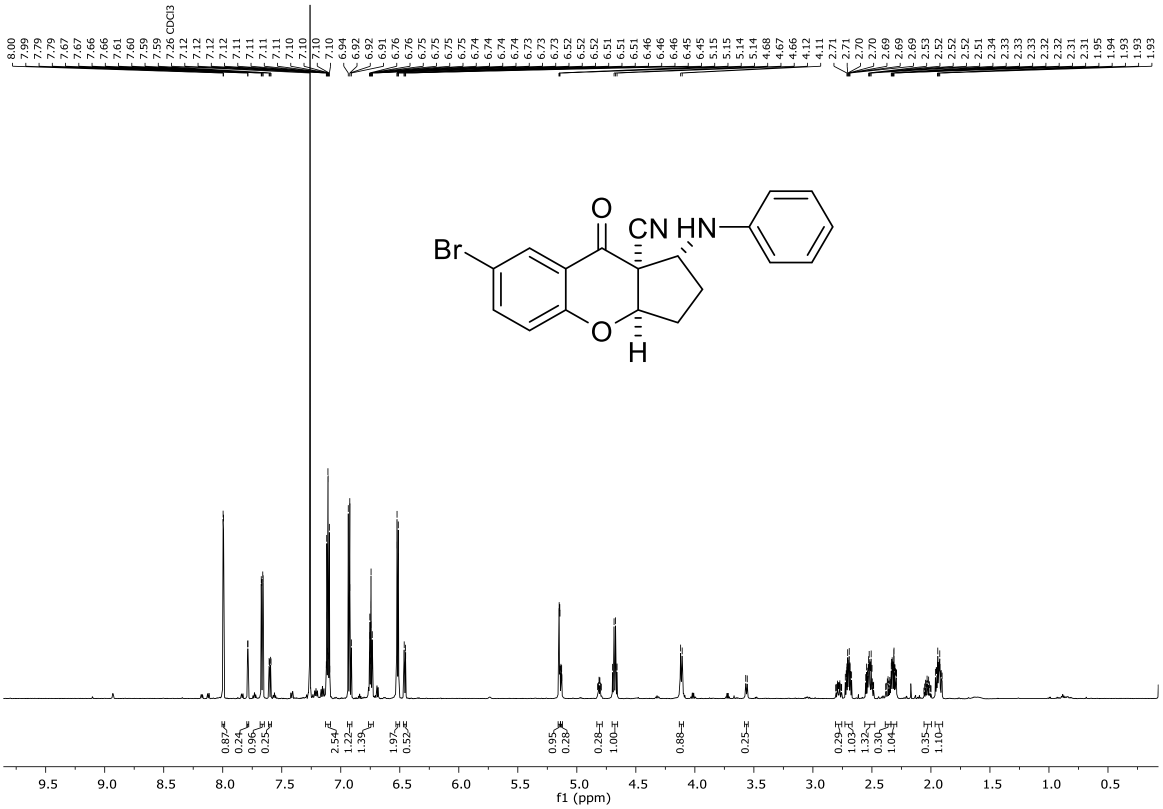

^{13}C{1H} NMR (176 MHz, $CDCl_3$)

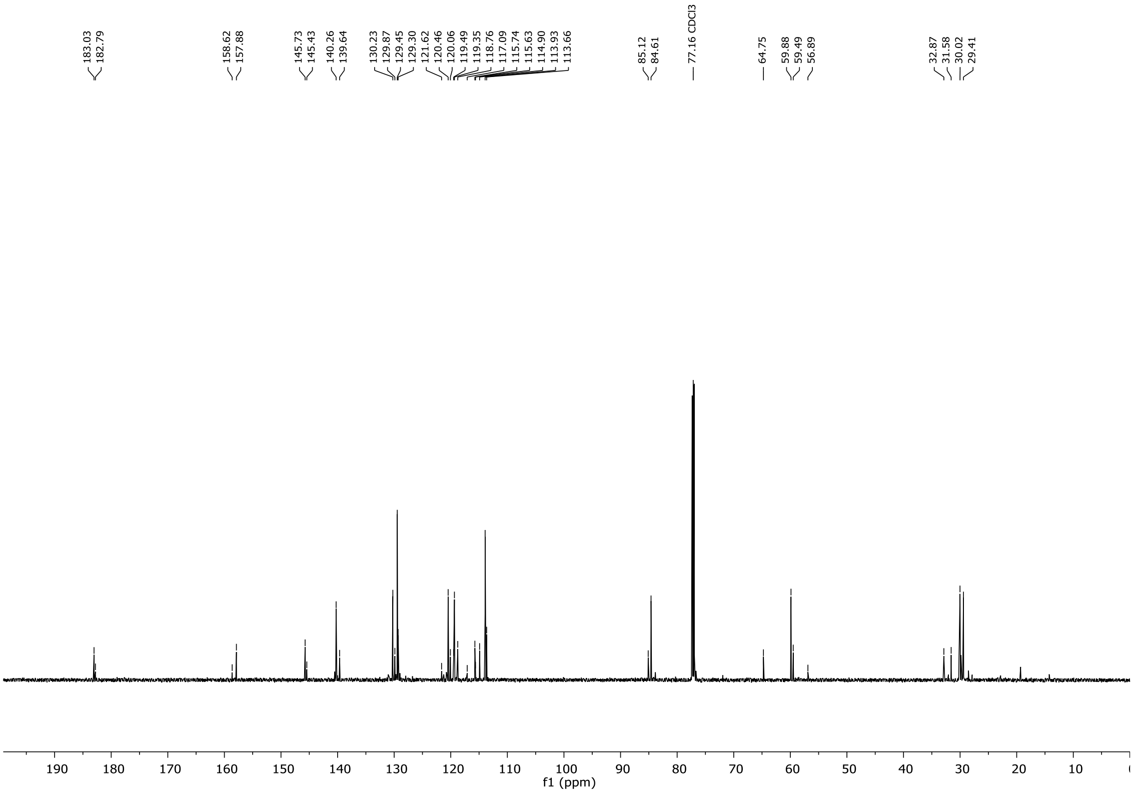

7-Chloro-9-oxo-1-(phenylamino)-1,2,3,3a,9,9a-hexahydrocyclopenta[*b*]chromene-9a-carbonitrile (*3fa*)

^{1}H NMR (700 MHz, CDCl$_3$)

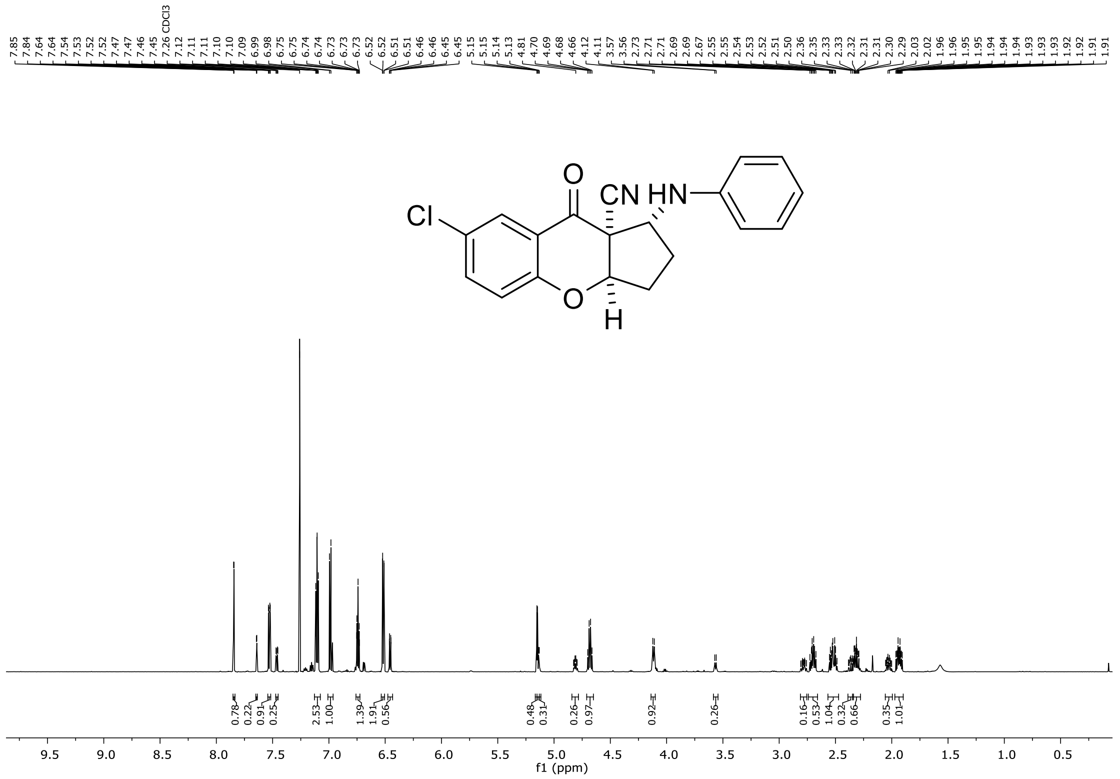

^{13}C{1H} NMR (176 MHz, CDCl$_3$)

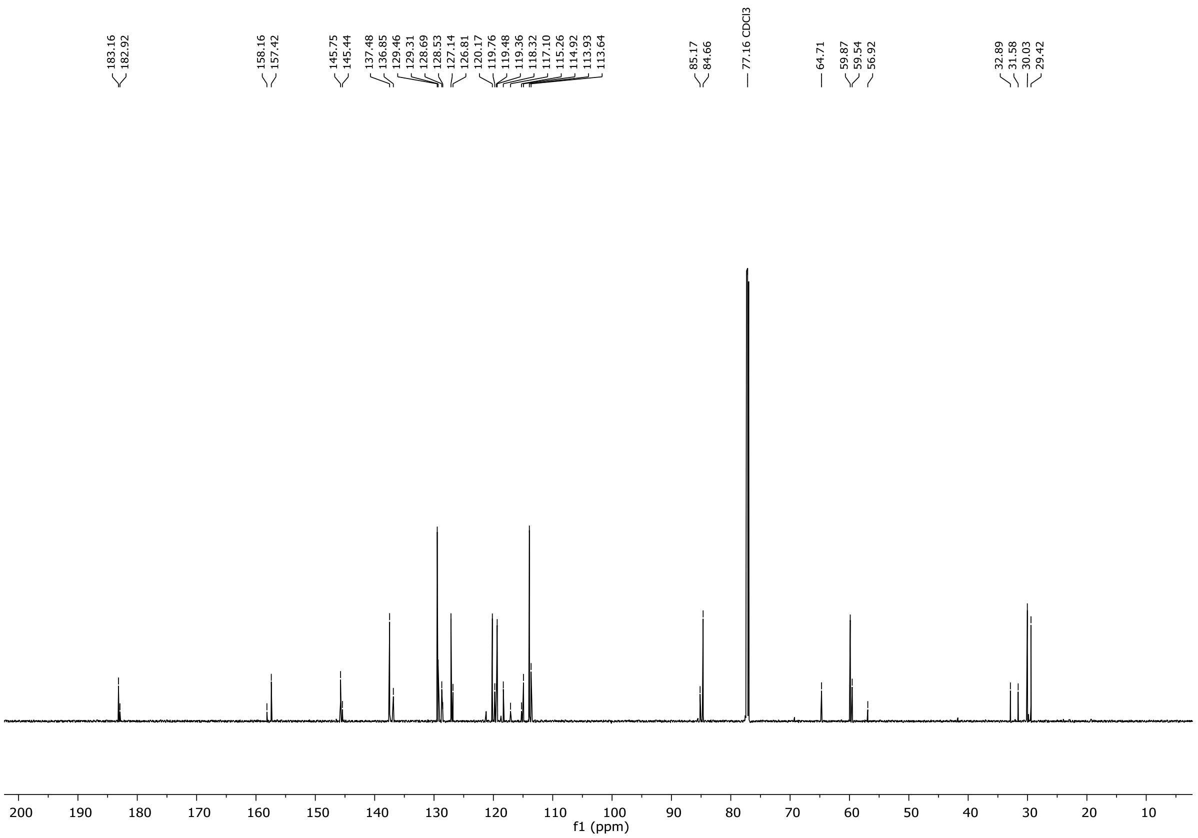

6-Methyl-9-oxo-1-(phenylamino)-1,2,3,3a,9,9a-hexahydrocyclopenta[*b*]chromene-9a-carbonitrile (*3ga*)

^{1}H NMR (700 MHz, CDCl$_3$)

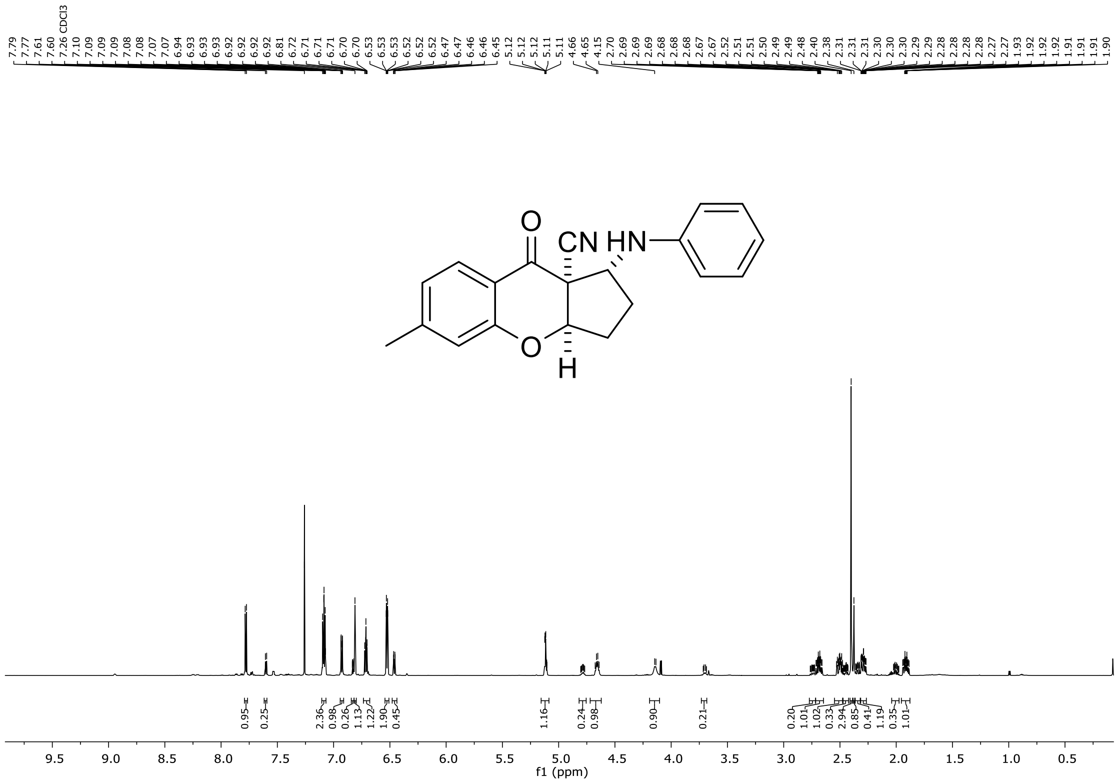

^{13}C{1H} NMR (176 MHz, CDCl$_3$)

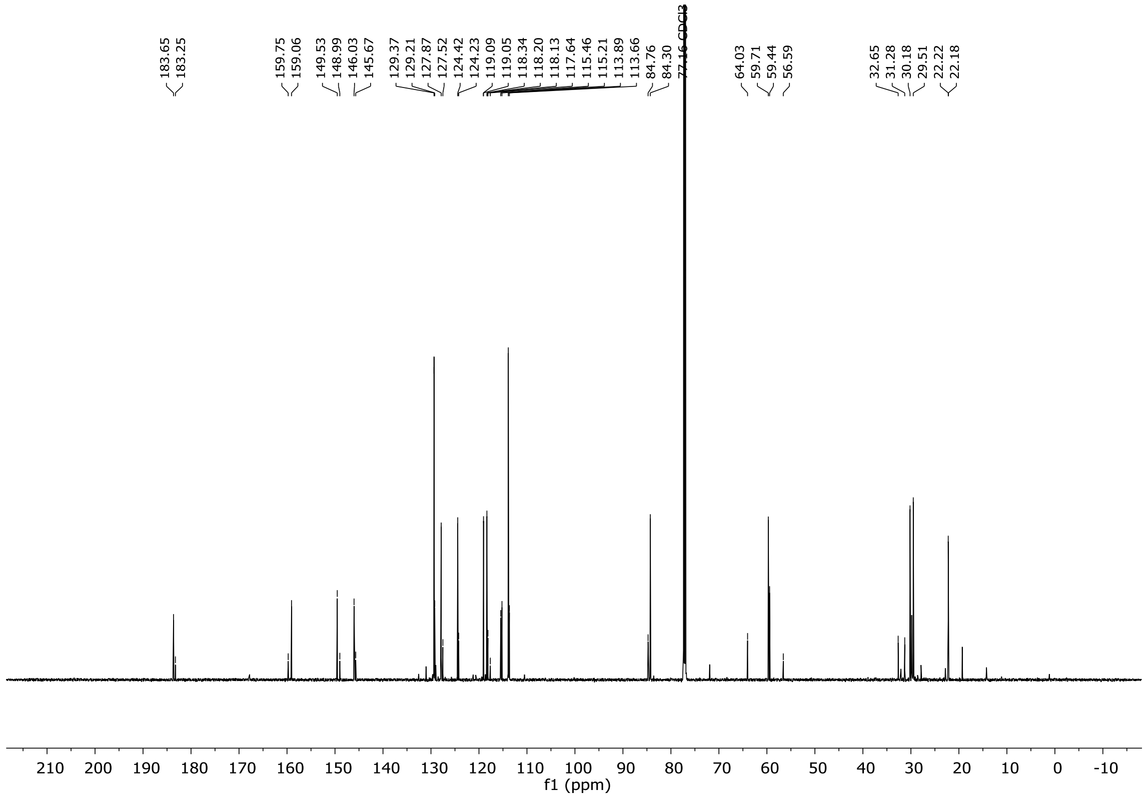

7-Methyl-9-oxo-1-(phenylamino)-1,2,3,3a,9,9a-hexahydrocyclopenta[*b*]chromene-9a-carbonitrile (*3ha*)

^{1}H NMR (700 MHz, CDCl$_3$)

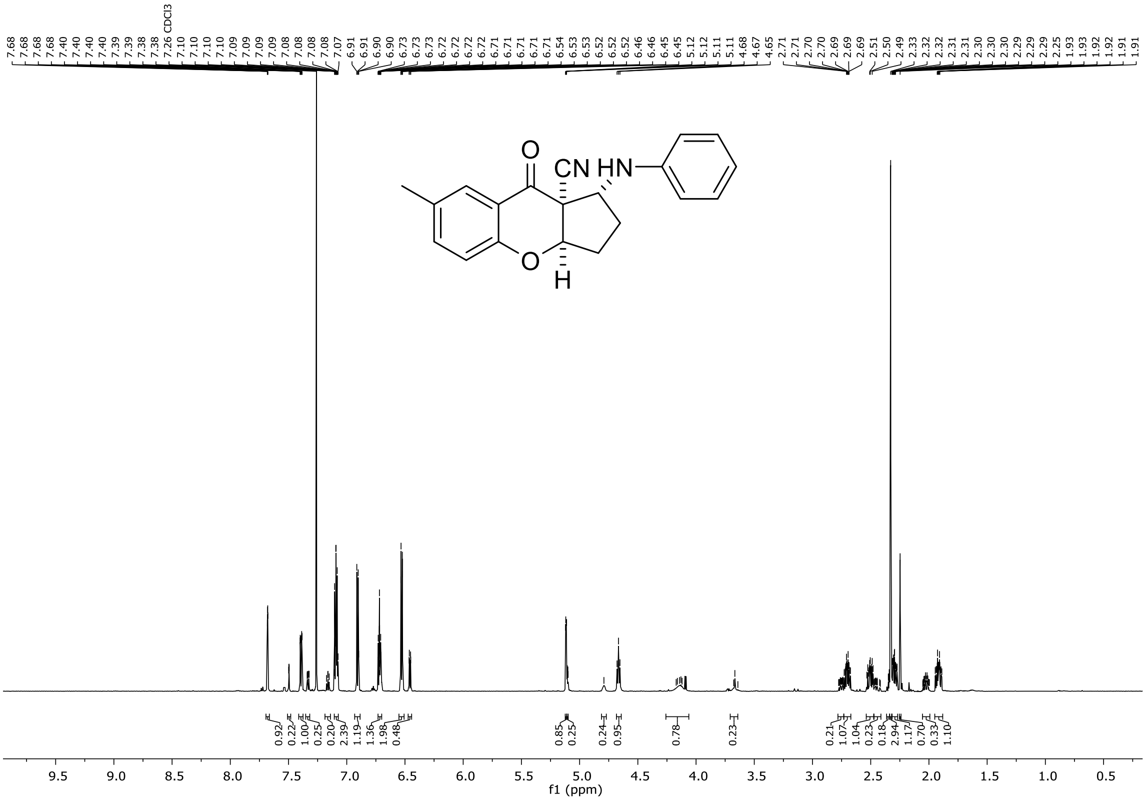

^{13}C{1H} NMR (176 MHz, CDCl$_3$)

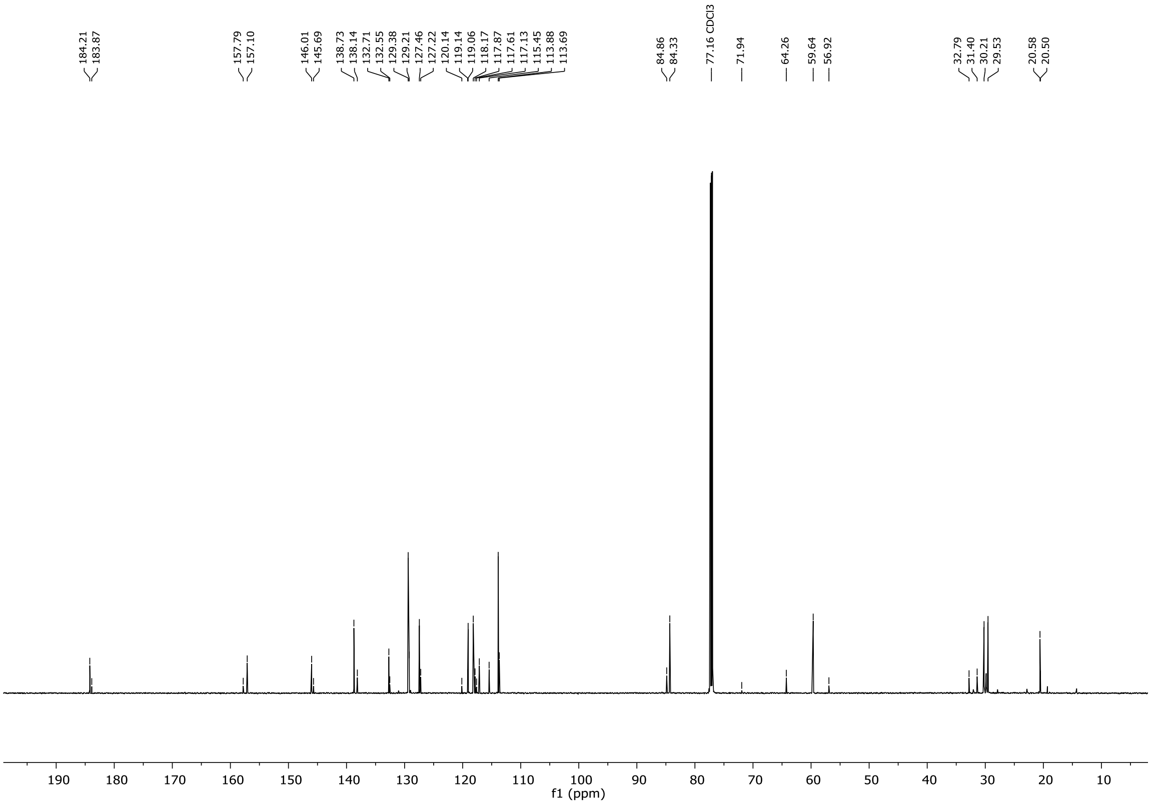

7-Chloro-6-methyl-9-oxo-1-(phenylamino)-1,2,3,3a,9,9a-hexahydrocyclopenta[*b*]chromene-9a-carbonitrile (*3ia*)

^{1}H NMR (700 MHz, CDCl$_3$)

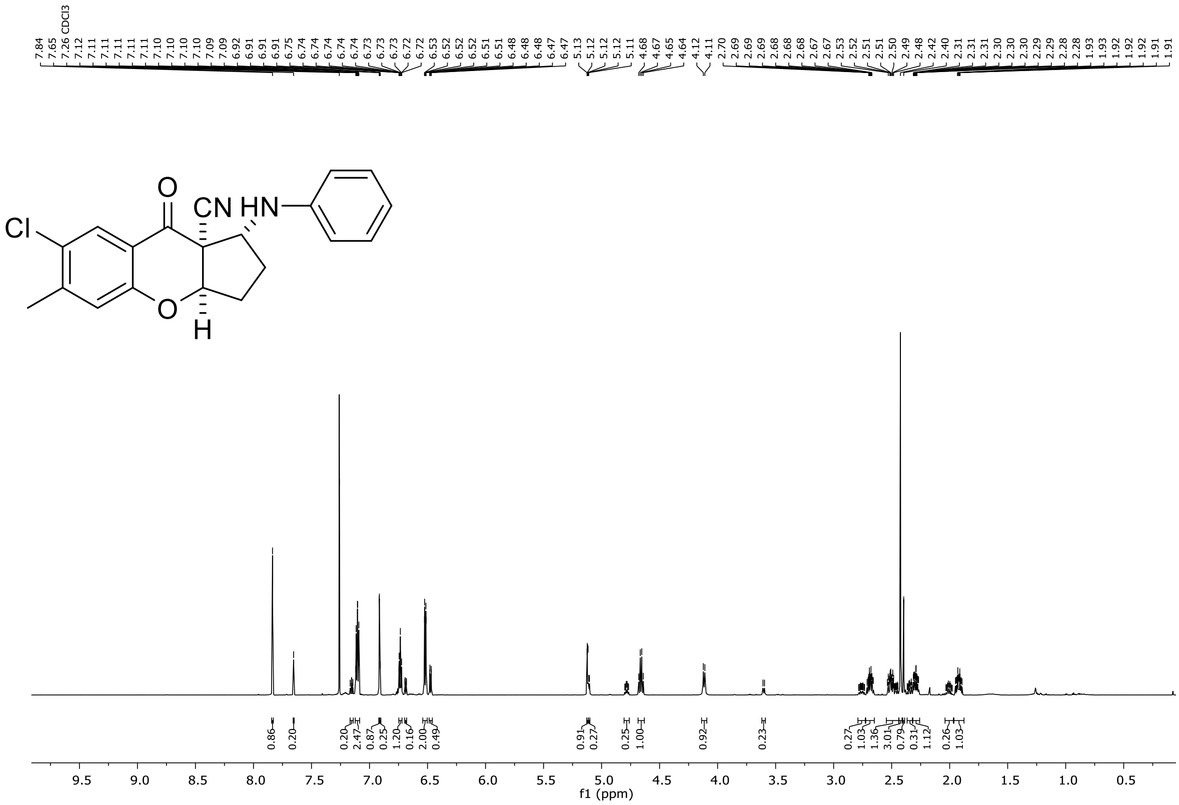

^{13}C{1H} NMR (176 MHz, CDCl$_3$)

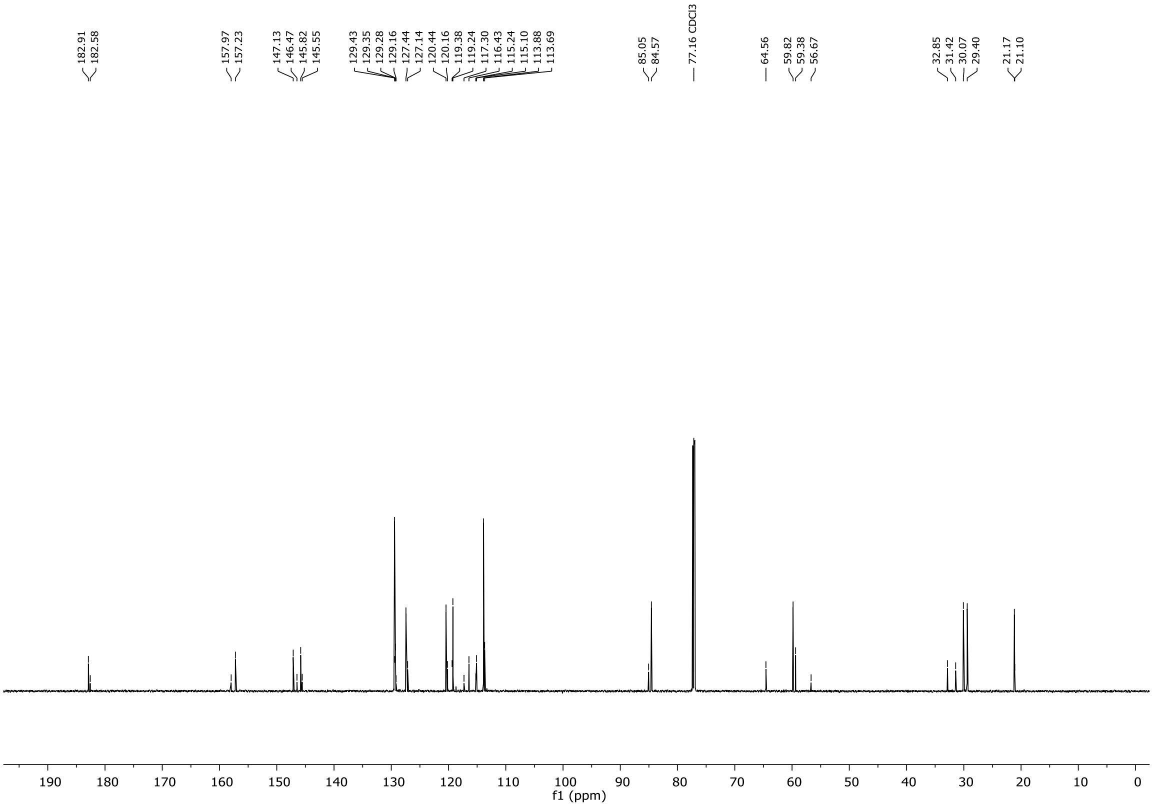

1-((4-Methoxyphenyl)amino)-9-oxo-1,2,3,3a,9,9a-hexahydrocyclopenta[*b*]chromene-9a-carbonitrile (*3ab*)

^{1}H NMR (700 MHz, CDCl$_3$)

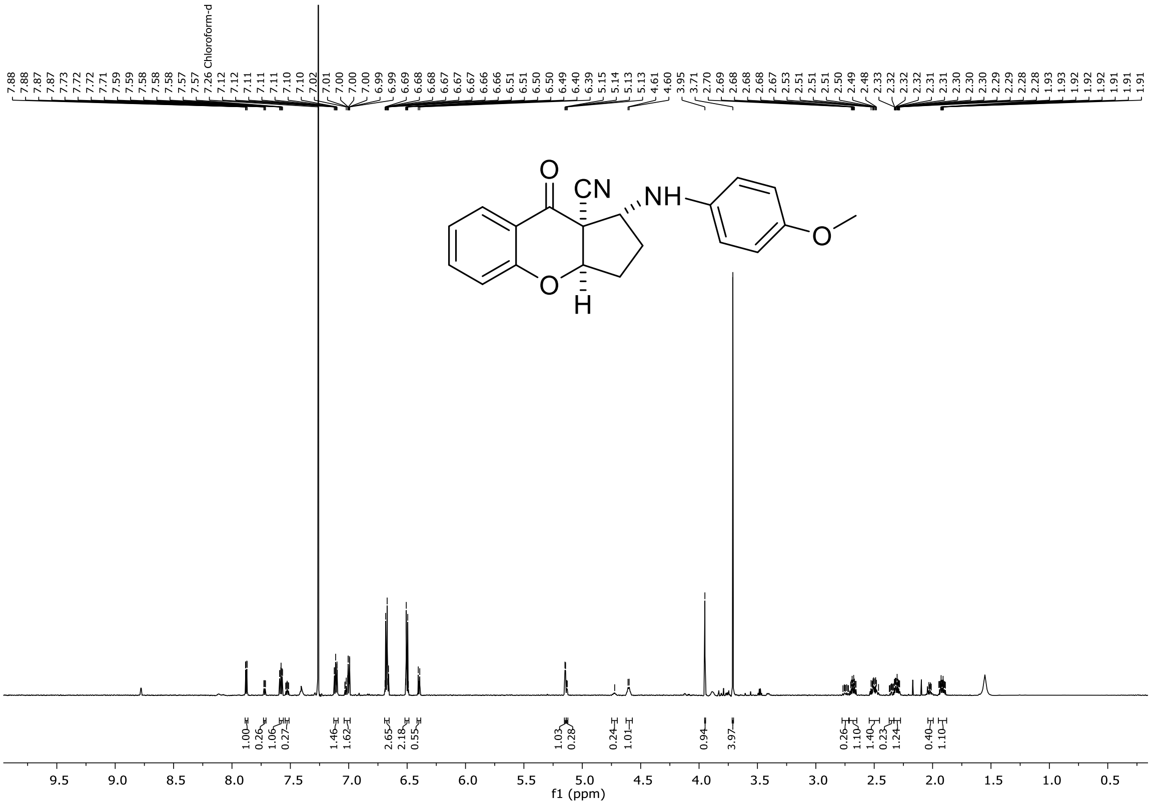

^{13}C{1H} NMR (176 MHz, CDCl$_3$)

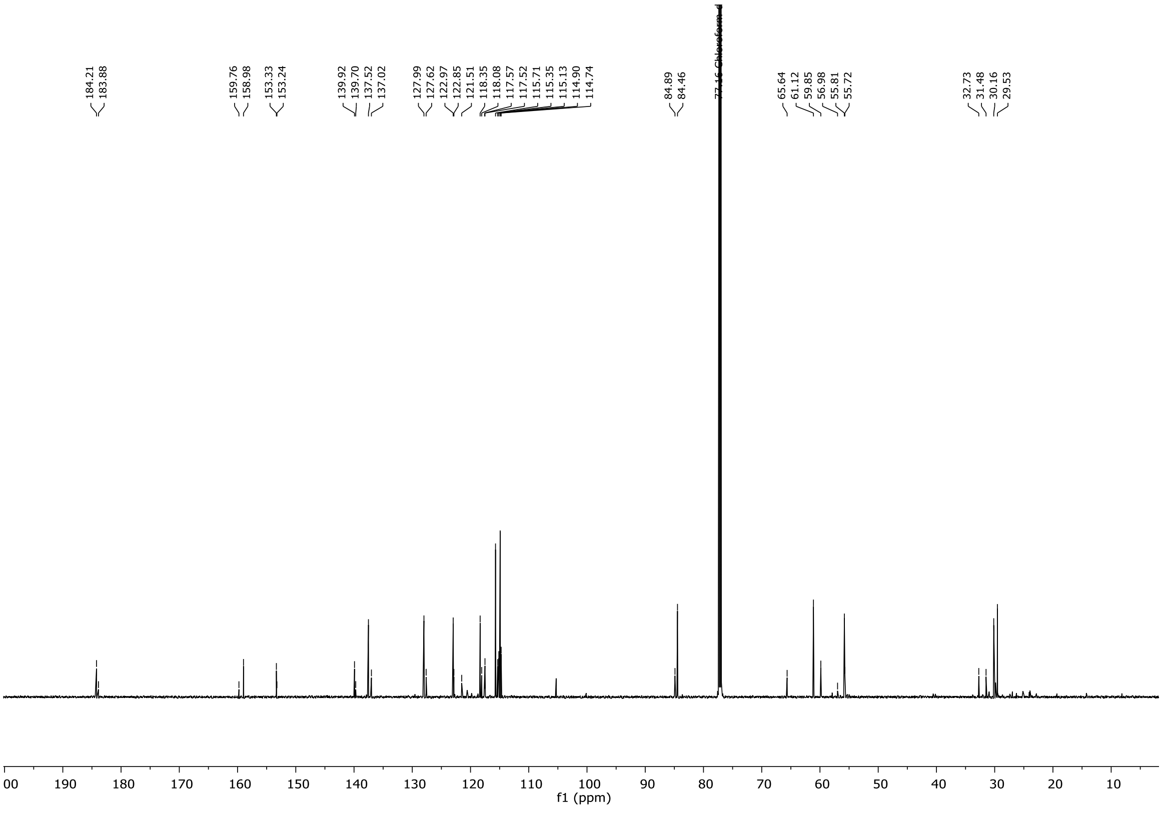

1-((4-(tert-butyl)phenyl)amino)-9-oxo-1,2,3,3a,9,9a-hexahydrocyclopenta[*b*]chromene-9a-carbonitrile (*3ac*)

^{1}H NMR (700 MHz, $CDCl_3$)

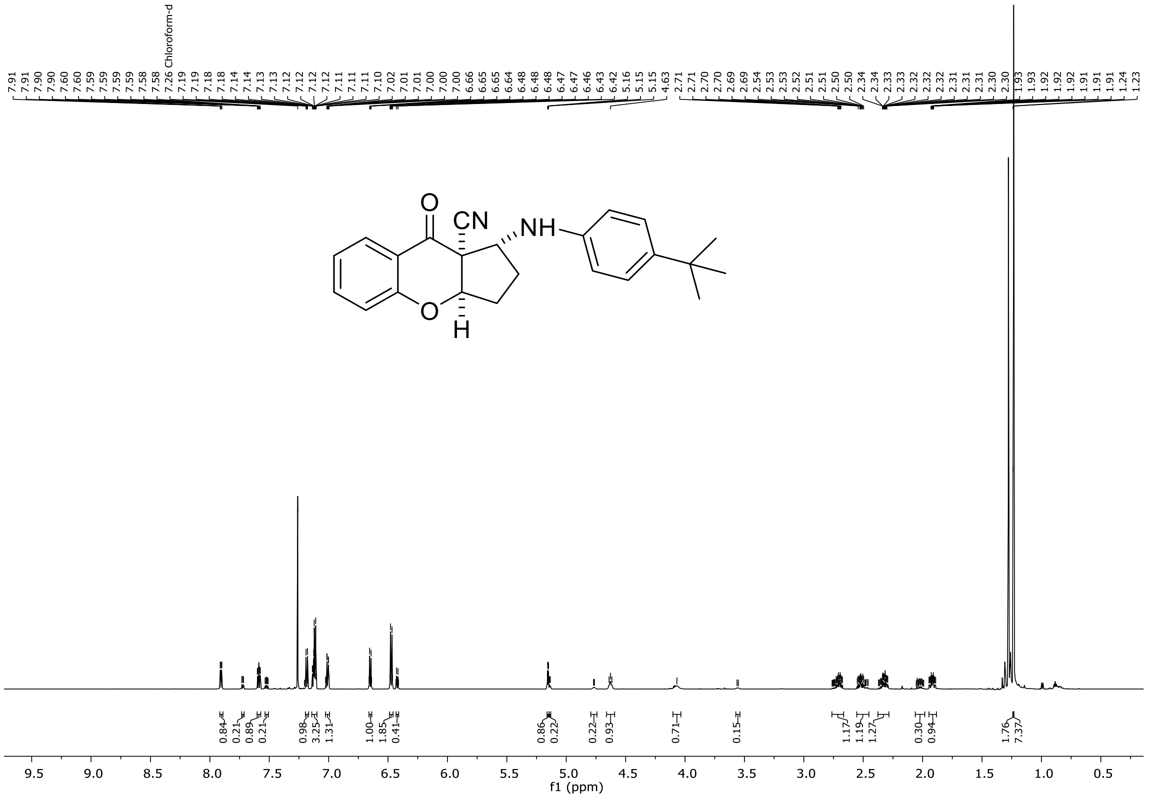

^{13}C{1H} NMR (176 MHz, $CDCl_3$)

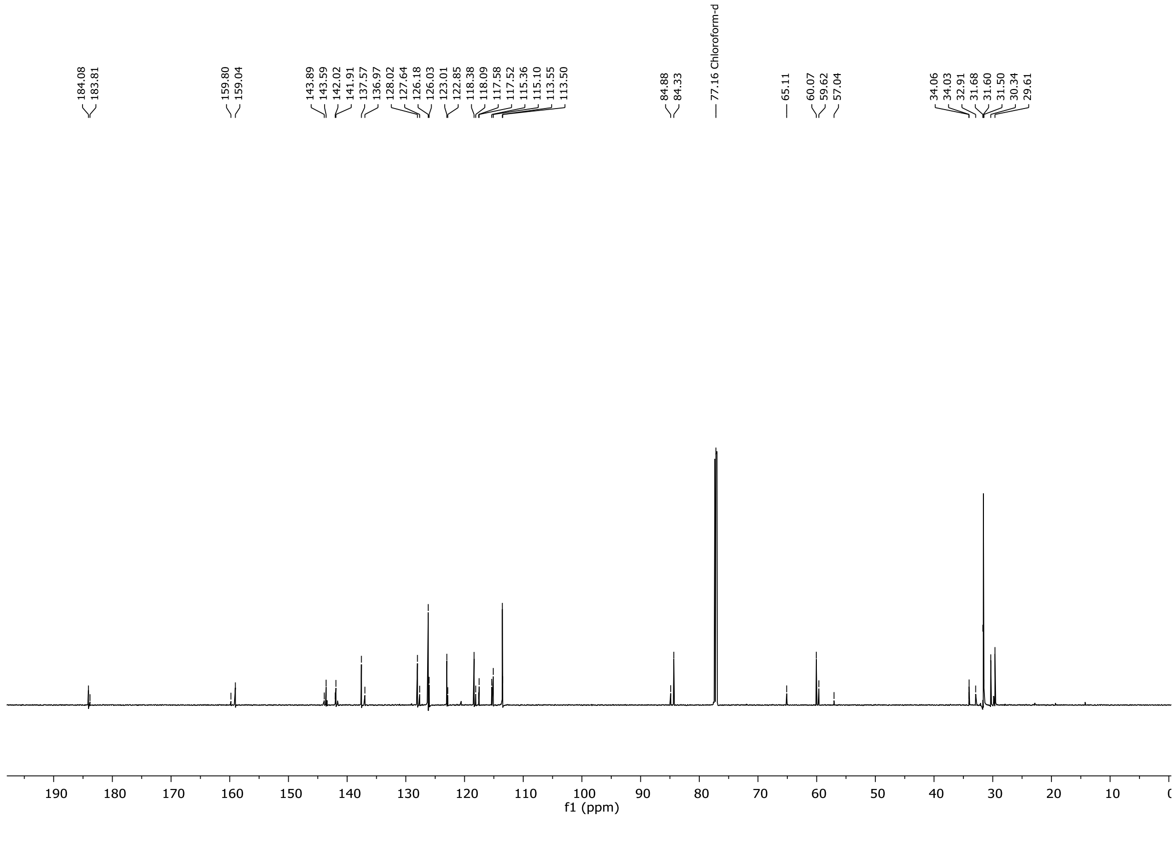

9-Oxo-1-((4-(trifluoromethyl)phenyl)amino)-1,2,3,3a,9,9a-hexahydrocyclopenta[*b*]chr-omene-9a-carbonitrile *(3ad*)

^{1}H NMR (700 MHz, CDCl$_3$)

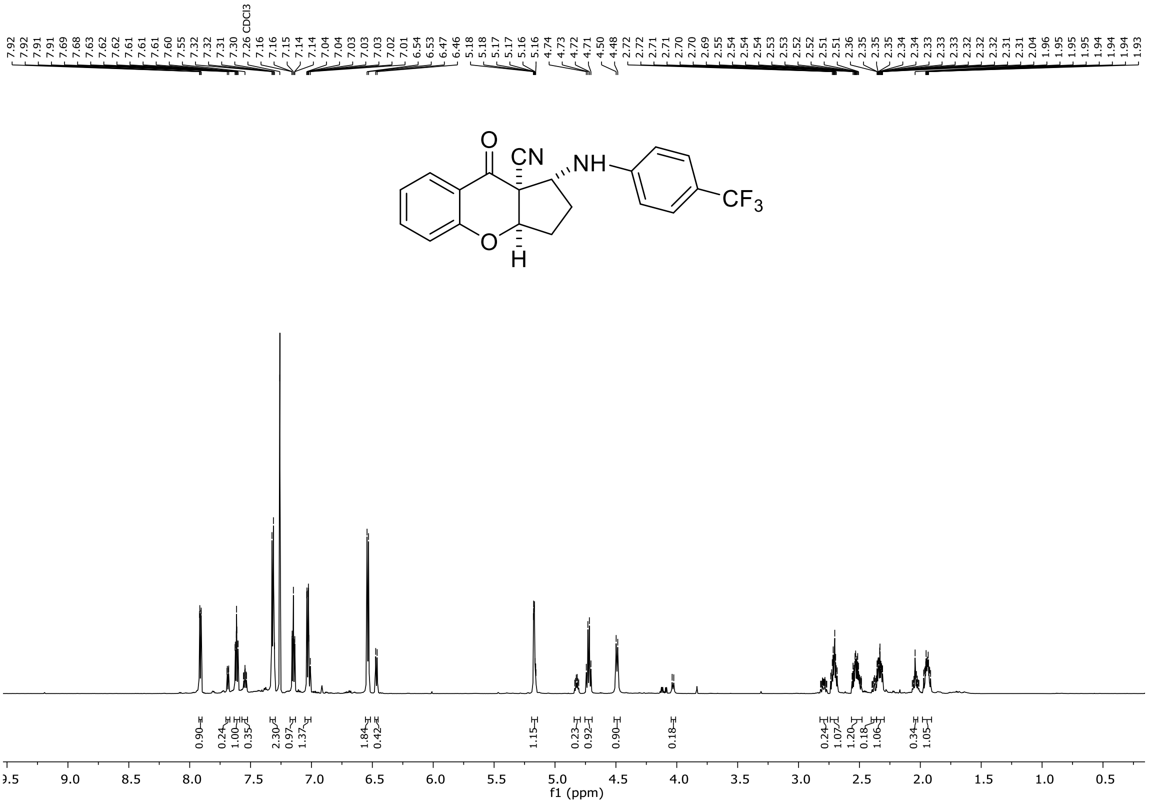

^{13}C{1H} NMR (176 MHz, CDCl$_3$)

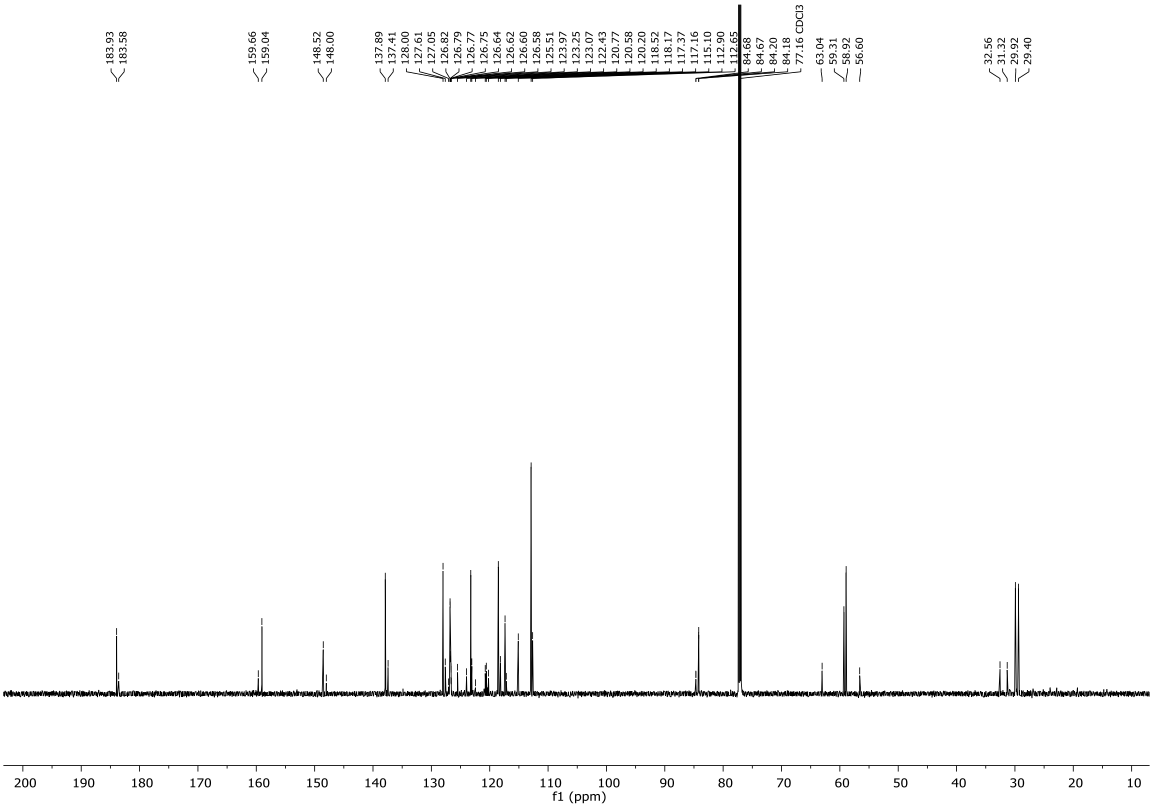

1-((3,5-Bis(trifluoromethyl)phenyl)amino)-9-oxo-1,2,3,3a,9,9a-hexahydrocyclopenta[*b*]-chromene-9a-carbonitrile *(3ae)*

^{1}H NMR (700 MHz, $CDCl_3$)

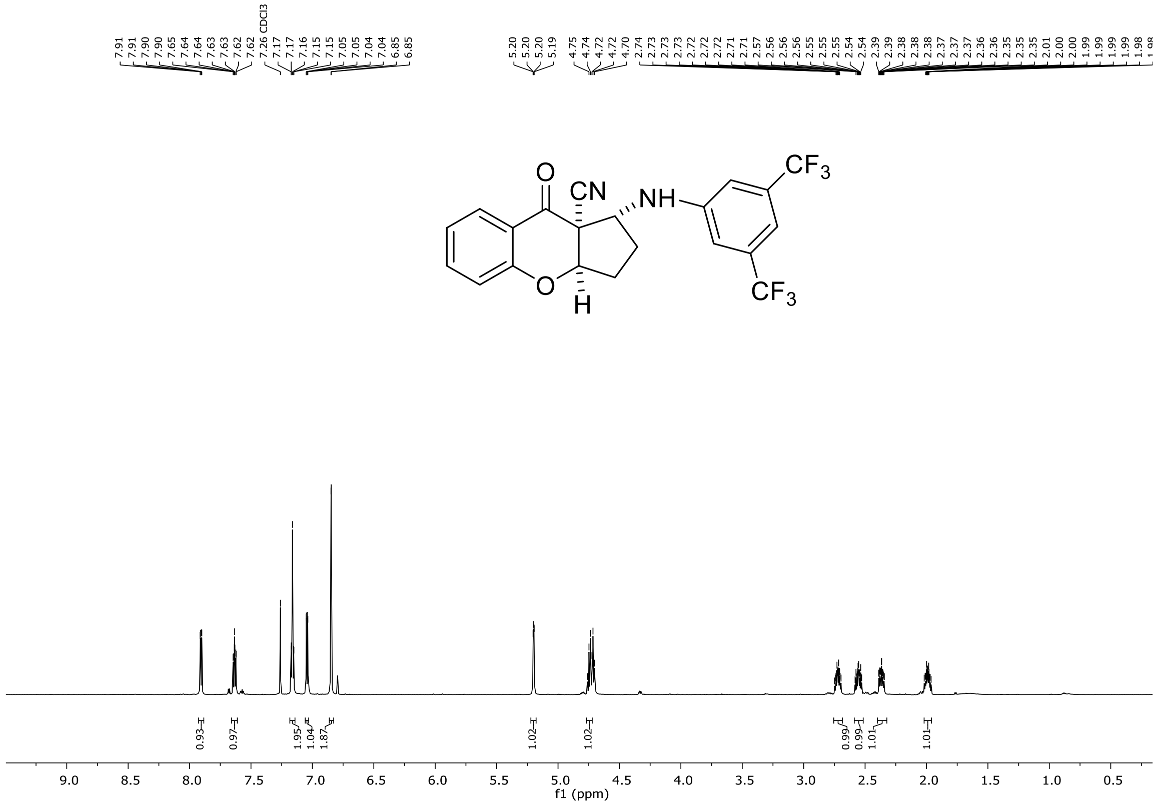

^{13}C{1H} NMR (176 MHz, $CDCl_3$)

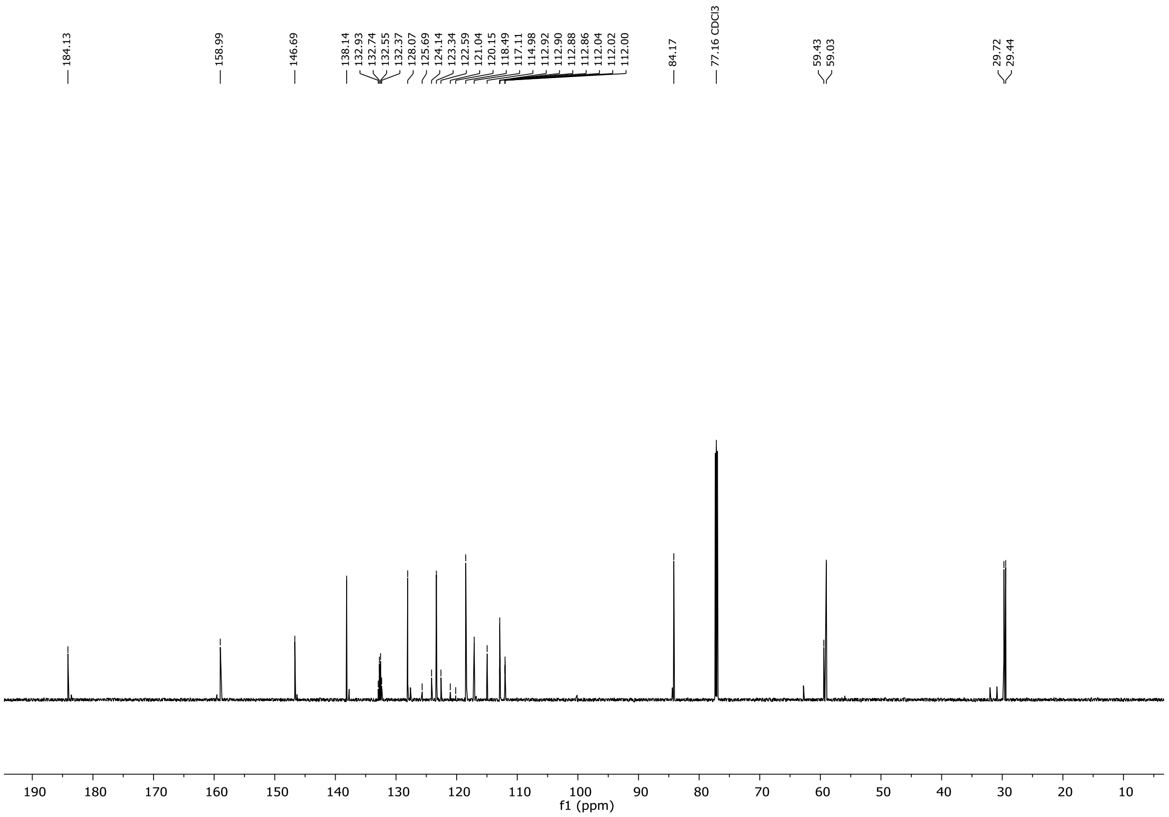

1-((2-chlorophenyl)amino)-9-oxo-1,2,3,3a,9,9a-hexahydrocyclopenta[*b*]chromene-9a-carbonitrile *(3af)*

^{1}H NMR (700 MHz, CDCl$_3$)

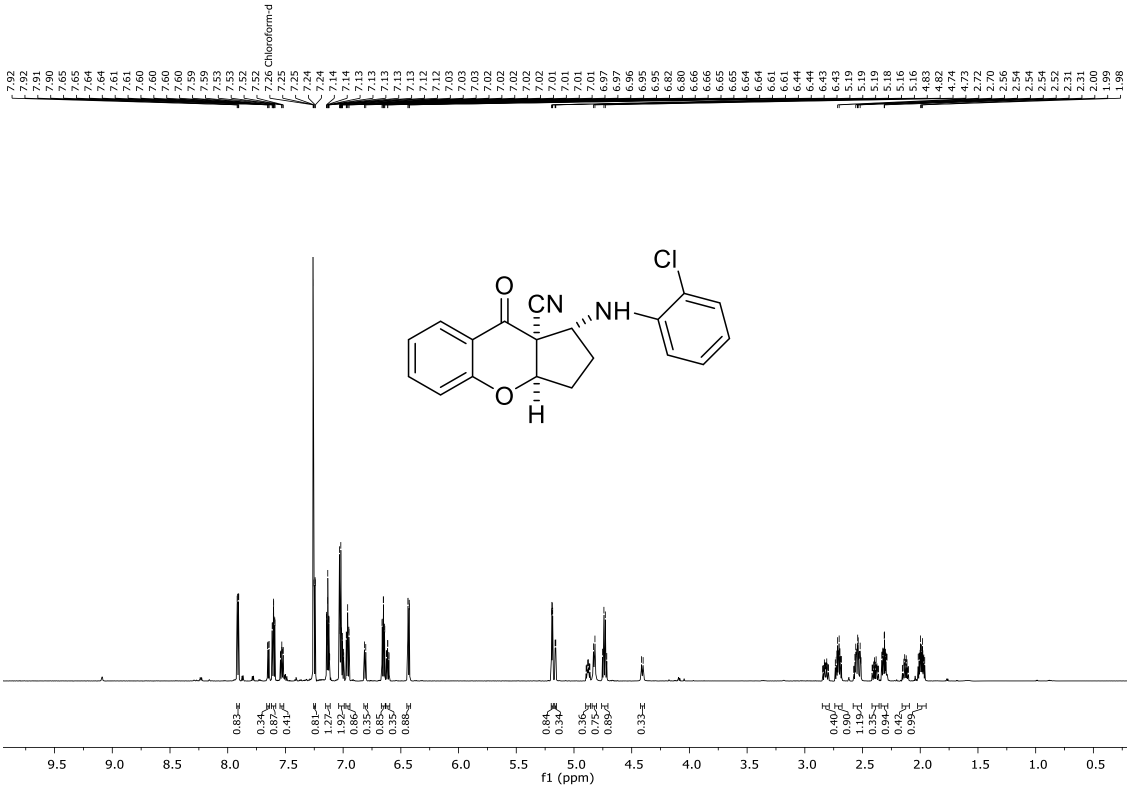

^{13}C{1H} NMR (176 MHz, CDCl$_3$)

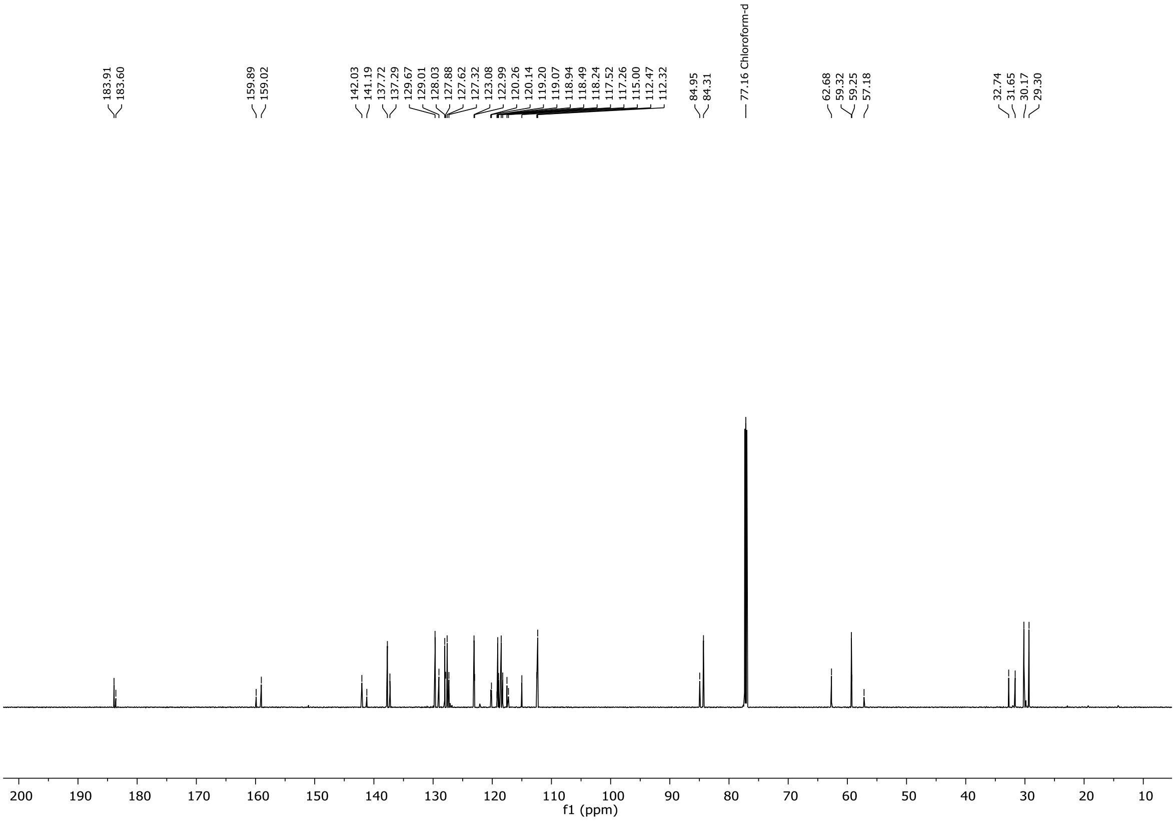

9-Hydroxy-6-methyl-1-(phenylamino)-1,2,3,3a,9,9a-hexahydrocyclopenta[*b*]chromene-9a-carbonitrile *(8ga)*

^{1}H NMR (700 MHz, CDCl$_3$)

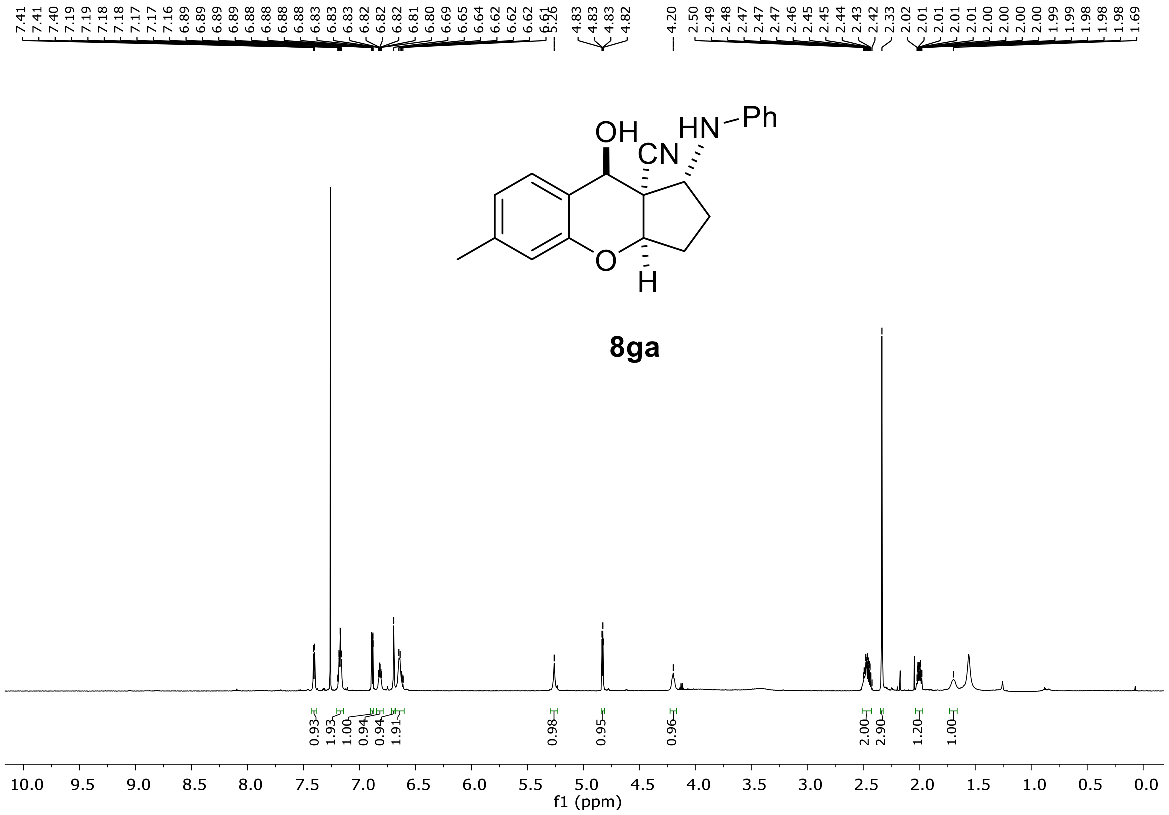

^{13}C{1H} NMR (176 MHz, CDCl$_3$)

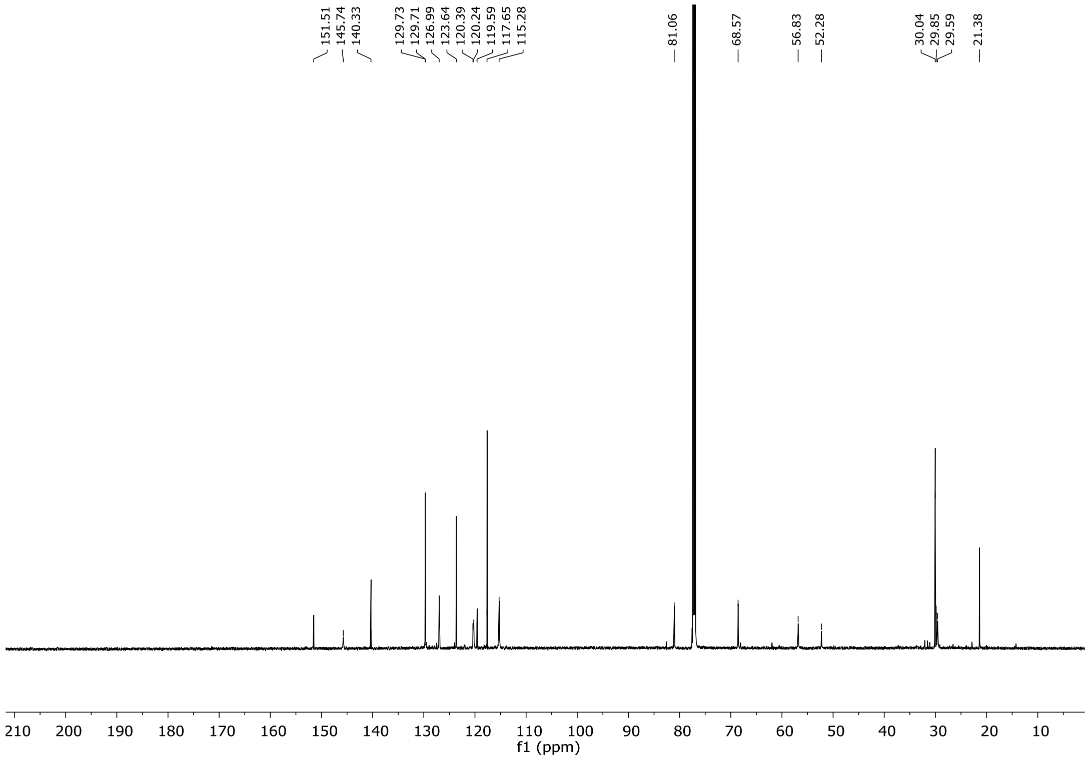

NOESY Experiment for **8ga**

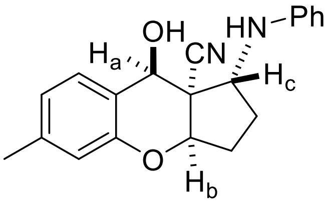

8ga

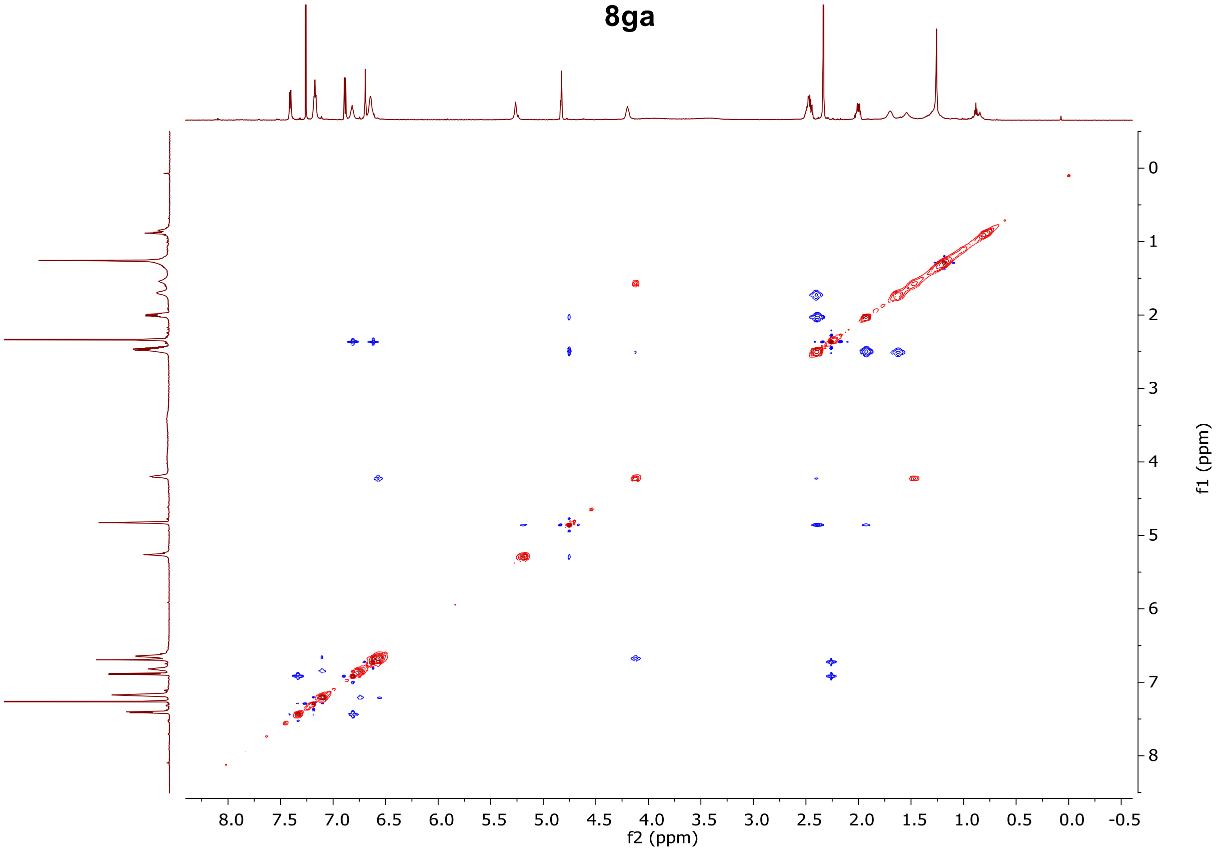

COSY Experiment for **8ga**

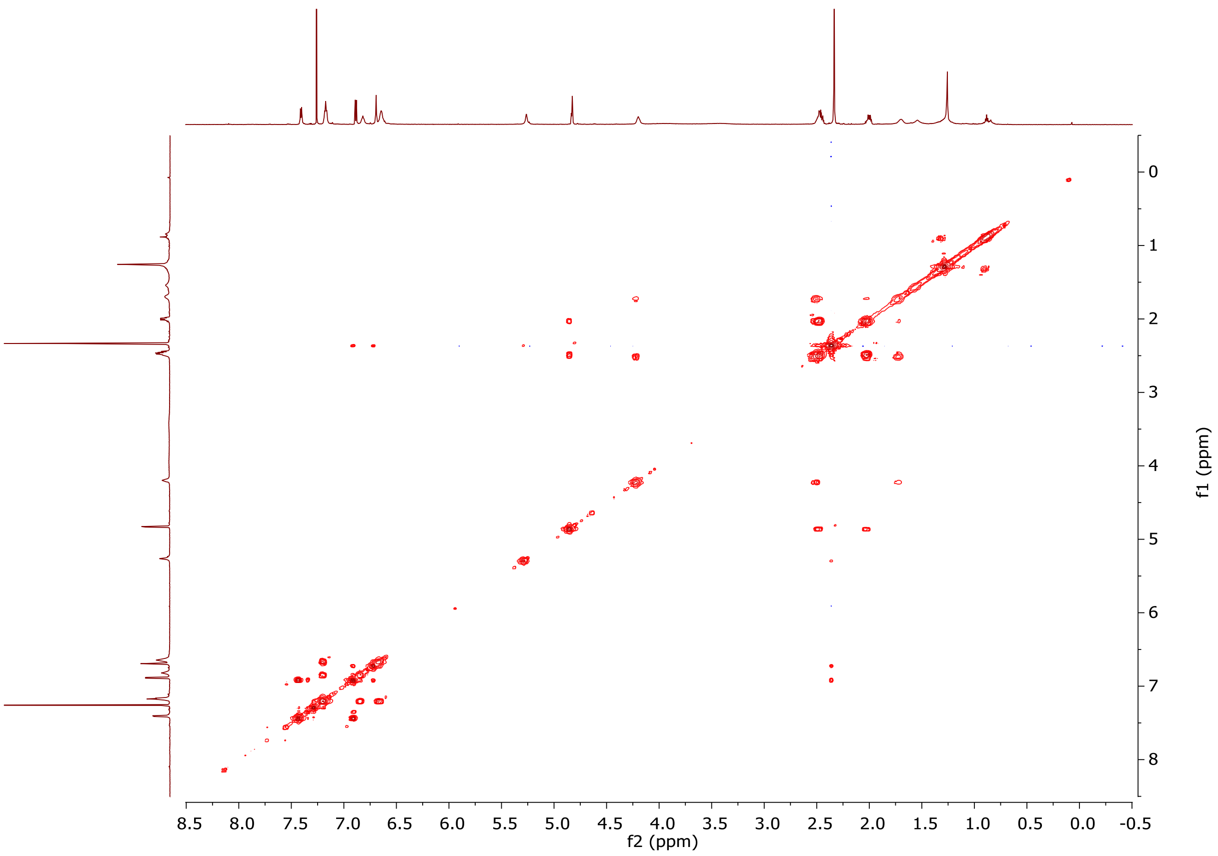

Supplement: Supplementary file 1 — jo3c02172_si_001.pdf [file jo3c02172_si_001.pdf]
